# A previously uncharacterized two-component signaling system in uropathogenic *Escherichia coli* coordinates protection against host-derived oxidative stress with activation of hemolysin-mediated host cell pyroptosis

**DOI:** 10.1371/journal.ppat.1010005

**Published:** 2021-10-15

**Authors:** Hongwei Gu, Xuwang Cai, Xinyang Zhang, Jie Luo, Xiaoyang Zhang, Xiao Hu, Wentong Cai, Ganwu Li

**Affiliations:** 1 Department of Veterinary Diagnostic and Production Animal Medicine, College of Veterinary Medicine, Iowa State University, Ames, Iowa, United States of America; 2 Central Laboratory, Nanjing Integrated Traditional Chinese and Western Medicine Hospital Affiliated with Nanjing University of Chinese Medicine, Nanjing, China; 3 State Key Laboratory of Agricultural Microbiology, College of Veterinary Medicine, Huazhong Agricultural University, Wuhan, China; 4 Key Laboratory of Veterinary Public Health of Ministry of Agriculture, State Key Laboratory of Veterinary Biotechnology, Harbin Veterinary Research Institute, Chinese Academy of Agricultural Sciences, Harbin, China; Tufts University, UNITED STATES

## Abstract

Uropathogenic *Escherichia coli* (UPEC) deploy an array of virulence factors to successfully establish urinary tract infections. Hemolysin is a pore-forming toxin, and its expression correlates with the severity of UPEC infection. Two-component signaling systems (TCSs) are a major mechanism by which bacteria sense environmental cues and respond by initiating adaptive responses. Here, we began this study by characterizing a novel TCS (C3564/C3565, herein renamed *orhK*/*orhR* for oxidative resistance and hemolysis kinase/regulator) that is encoded on a UPEC pathogenicity island, using bioinformatic and biochemical approaches. A prevalence analysis indicates that *orhK*/*orhR* is highly associated with the UPEC pathotype, and it rarely occurs in other *E*. *coli* pathotypes tested. We then demonstrated that OrhK/OrhR directly activates the expression of a putative methionine sulfoxide reductase system (C3566/C3567) and hemolysin (HlyA) in response to host-derived hydrogen peroxide (H_2_O_2_) exposure. OrhK/OrhR increases UPEC resistance to H_2_O_2_ *in vitro* and survival in macrophages in cell culture via C3566/C3567. Additionally, OrhK/OrhR mediates hemolysin-induced renal epithelial cell and macrophage death via a pyroptosis pathway. Reducing intracellular H_2_O_2_ production by a chemical inhibitor impaired OrhK/OrhR-mediated activation of *c3566-c3567* and *hlyA*. We also uncovered that UPEC links the two key virulence traits by cotranscribing the *c3566-c3567* and *hlyCABD* operons. Taken together, our data suggest a paradigm in which a signal transduction system coordinates both bacterial pathogen defensive and offensive traits in the presence of host-derived signals; and this exquisite mechanism likely contributes to hemolysin-induced severe pathological outcomes.

## Author summary

Uropathogenic *Escherichia coli* (UPEC) is the primary cause of urinary tract infections, and approximately half of UPEC isolates produce a pore-forming toxin, hemolysin. Clinically, hemolysin carriage is associated with severe pathology and symptoms during UPEC infections. However, overexpression of hemolysin can be detrimental to UPEC colonization. Therefore, fine-tuning of hemolysin expression in response to *in vivo*-relevant signals is critical for optimal UPEC fitness in the urinary tract. In this study, we describe a virulence strategy employed by UPEC, i.e., the bacteria use a two-component signaling (TCS) system to coordinate oxidative stress resistance and hemolysin-mediated pyroptosis of host cells in response to host-derived oxidative signals. The TCS achieves this coordination by cotranscribing genes encoding the oxidative stress resistance and the hemolysin. As a result, UPEC is able to link defense to offense, and this exquisite virulence mechanism likely contributes to UPEC fitness *in vivo* and hemolysin-induced severe pathological outcomes.

## Introduction

Uropathogenic *Escherichia coli* (UPEC) cause most urinary tract infections (UTIs) worldwide. Cystitis and pyelonephritis are common clinical manifestations of UTIs, and in some severe cases, bacteremia occurs [1,2]. Upon entry into the urethral opening, UPEC can ascend through the urethra and finally arrive at the bladder, where they can colonize and establish infection [3]. Immune cells, such as macrophages and neutrophils, function as the first-line barriers to UPEC infection, usually by engulfing and killing the bacteria with reactive oxygen (ROS, e.g., H_2_O_2_), chlorine (RCS, e.g., HOCl) and nitrogen species (RNS, e.g., NO) [4–6]. However, pathogenic bacteria can resist hostile immune killing, which can be deemed defensive activity. OxyR is a conserved H_2_O_2_-sensing transcriptional regulator in *E*. *coli* [7], and it protects the cell from oxidative stress by controlling a regulon of nearly 40 genes, for instance genes encoding alkyl hydroperoxide reductase AhpCF and catalase KatG [8]. Other defense systems in UPEC include the KatE catalase that decomposes H_2_O_2_ [9], and RpoS [10] regulator that elicits global response to withstand oxidative stress. As another strategy for survival, many bacterial pathogens encode toxins that can damage host cells in various ways [11,12], which can be deemed offensive activity. Thus, an effective and efficient coordination of defense and offense during infection is conceivably an important fitness factor for pathogens.

UPEC form a very diverse group of bacteria possessing a large repertoire of virulence factors [13], including hemolysin. Hemolysin is a prototypic alpha pore-forming toxin that is carried by approximately half of UPEC clinical isolates, with an even greater percentage in pyelonephritis isolates. Clinically, hemolysin carriage is associated with severe symptoms, especially kidney damage [14,15]. Hemolysin has been proposed to function differentially over a gradient of concentrations. At high concentrations, it can form pores in membranes of various host cell types, including red blood cells (RBCs), epithelial cells and leukocytes, resulting in cell lysis [16–18]. However, at low (sublytic) concentrations, hemolysin can either alter cell functions or induce cell death. For example, in UPEC strain UTI89, sublytic doses of hemolysin can activate host proteases, especially mesotrypsin, resulting in degradation of paxillin that aids in host cell membrane damage and further detachment of the cell [19]. One study reported that hemolysin from UTI89 activates a caspase-1/caspase-4-dependent death pathway, most likely pyroptosis, in human bladder epithelial cells [20]. In addition, hemolysin produced by CP9 isolated from a sepsis patient’s blood has been found to mediate apoptosis and necrosis of human neutrophils [17]. Despite the well-documented toxic functions of hemolysin, in-depth studies on its regulation are very limited. Reported regulators include CpxR [20], FNR [21], RfaH [22] and BarA/UvrY [23], and several other factors have been identified in genetic screens, including Cof, which is a phosphatase [16]; LPS biosynthesis gene products; and DnaKJ chaperones [24]. Importantly, the settings in which these factors contribute are insufficiently studied.

Two-component signaling systems (TCSs), which are composed of a membrane-bound histidine kinase (HK) sensor and a cytoplasmic response regulator (RR), have been implicated in regulating bacterial responses to a variety of signals and stimuli, such as nutrients and small-molecule signals. Recognition of physical or chemical signals by the HK sensor domain typically triggers modulation of HK autophosphorylation activity. The phosphoryl group is then transferred to the RR, which is usually a DNA-binding protein that acts to alter gene expression [25,26]. Our group have previously revealed that UPEC-associated TCS KguS/R, which responds to the presence of α-ketoglutarate (KG), a particularly abundant metabolite within renal proximal tubule cells, stimulates the expression of KG utilization genes [27]. As UPEC reside in distinct host environments compared to enteric *E*. *coli* [28], it seems plausible that UPEC carry a particular set of TCSs for environmental adaptation and virulence.

In this study, our group identified a previously uncharacterized genomic island-encoded TCS, *c3564*/*c3565*, that is highly associated with UPEC. In response to H_2_O_2_ exposure *in vitro* and within macrophages, C3564/C3565 directly activates the expression of *c3566*-*c3567*, which encodes a putative methionine sulfoxide (MetSO) reductase system (Msr) that protects the bacteria from oxidative insults, increasing survival in oxidative environments and in host cells. In addition, C3564/C3565 promotes hemolysin-mediated host cell pyroptosis in the presence of H_2_O_2_. Mechanistically, C3564/C3565 coordinates oxidative resistance and toxin production by cotranscribing the *c3566*-*c3567* and *hlyCABD* operons. Overall, our study provides a paradigm in which a regulatory system links together defensive and offensive activities in a bacterial pathogen.

## Results

### C3564 and C3565 constitute a cognate TCS

**Fig 1:**
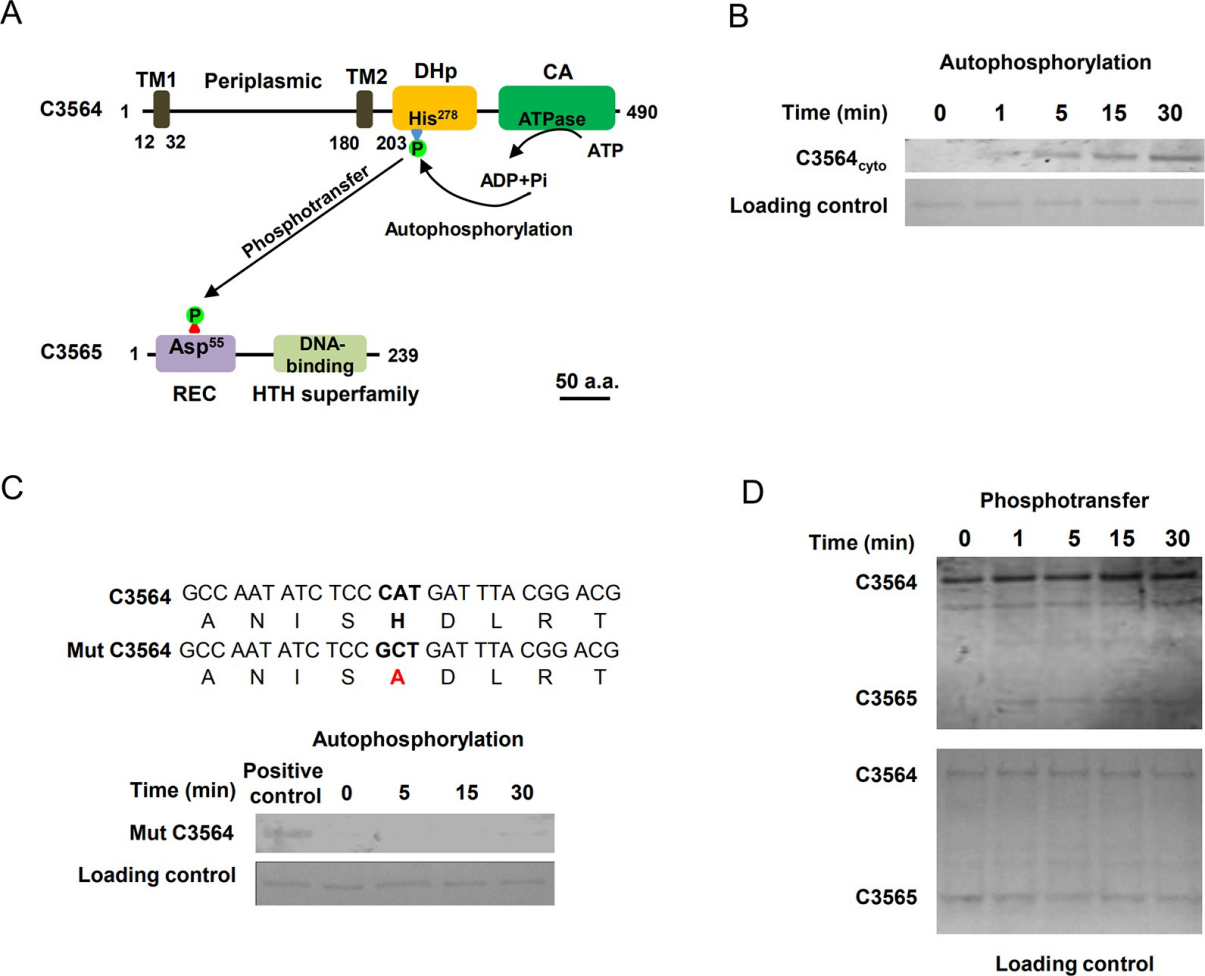
Identification of a two-component signaling system, C3564/C3565, in UPEC CFT073. **(A)** Domain architectures of C3564 and C3565. The numbers indicate the amino acid positions. The domains were predicted with the InterPro database. Abbreviations: TM1 and TM2, two transmembrane helices; DHp, dimerization and histidine phosphorylation domain containing His278 for autophosphorylation; CA, catalytic ATPase domain; REC, receiver domain containing the putative aspartate residue for receiving the phosphoryl group; HTH, helix-turn-helix domain for DNA binding. The scale bar indicates 50 amino acids (a.a.). **(B)** *In vitro* analysis of C3564 autophosphorylation. The purified cytoplasmic portion of C3564 was incubated with ATP for various amounts of time, and phosphorylated proteins were detected using the pIMAGO-biotin phosphoprotein detection method. **(C)** Mutation of His278 abolished C3564 self-phosphorylation. Mut C3564 is a variant of C3564 carrying a His278Ala mutation, and the positive control is the wild-type C3564 protein. **(D)** *In vitro* transphosphorylation of C3565 by phosphorylated C3564. The autophosphorylated form of C3564 and purified C3565 protein were mixed at equimolar concentrations and then incubated at 37°C for the indicated amounts of time. The reaction mixture was directly subjected to SDS-PAGE and detected by the pIMAGO-biotin phosphoprotein detection method. These experiments were repeated at least twice, and representative images are shown.

In searching for previously uncharacterized TCSs in pathogenic *E*. *coli*, we found that *c3564* and *c3565*, which are located in the *pheV* genomic island in UPEC CFT073, are predicted to encode a TCS. C3564 is predicted to be an HK that contains a periplasmic domain flanked by a transmembrane helix on each side, a dimerization/phosphoacceptor (HisKA) domain containing a conserved autophosphorylation site (histidine, His278), and an ATPase domain (Fig 1A). C3565 is predicted to be an OmpR subfamily RR possessing a CheY-like receiver domain containing an aspartate residue (Asp55) presumed to accept a phosphoryl group from an HK, as well as a winged helix-turn-helix (HTH) DNA-binding domain (Fig 1A).

To examine whether C3564 and C3565 constitute a cognate TCS, autophosphorylation and transphosphorylation assays were performed with purified recombinant proteins. C3564 was expressed as a cytoplasmic portion (C3564_cyto_)-GST fusion protein, and C3565 as a His_6_-C3565 fusion protein. Autophosphorylation occurred rapidly (at 1 min) and increased as the incubation was prolonged (Fig 1B). In contrast, when a mutant form of C3564 in which the conserved His278 was changed to alanine (His278Ala) was tested, no autophosphorylation was detected within the 30 min time frame of the assay (Fig 1C), suggesting that His278 is involved in accepting the phosphoryl group. A transphosphorylation assay was performed using ATP-saturated C3564 coincubated with C3565. We observed markedly increased levels of C3565 phosphorylation within 1 min compared to the levels for the control protein at 0 min (Fig 1D), indicating that C3565 can be transphosphorylated by C3564. Together, these results suggest that C3564 and C3565 constitute a cognate TCS.

### *c3564* and *c3565* are highly associated with UPEC and promote host cell death during infection in cell culture models

**Fig 2:**
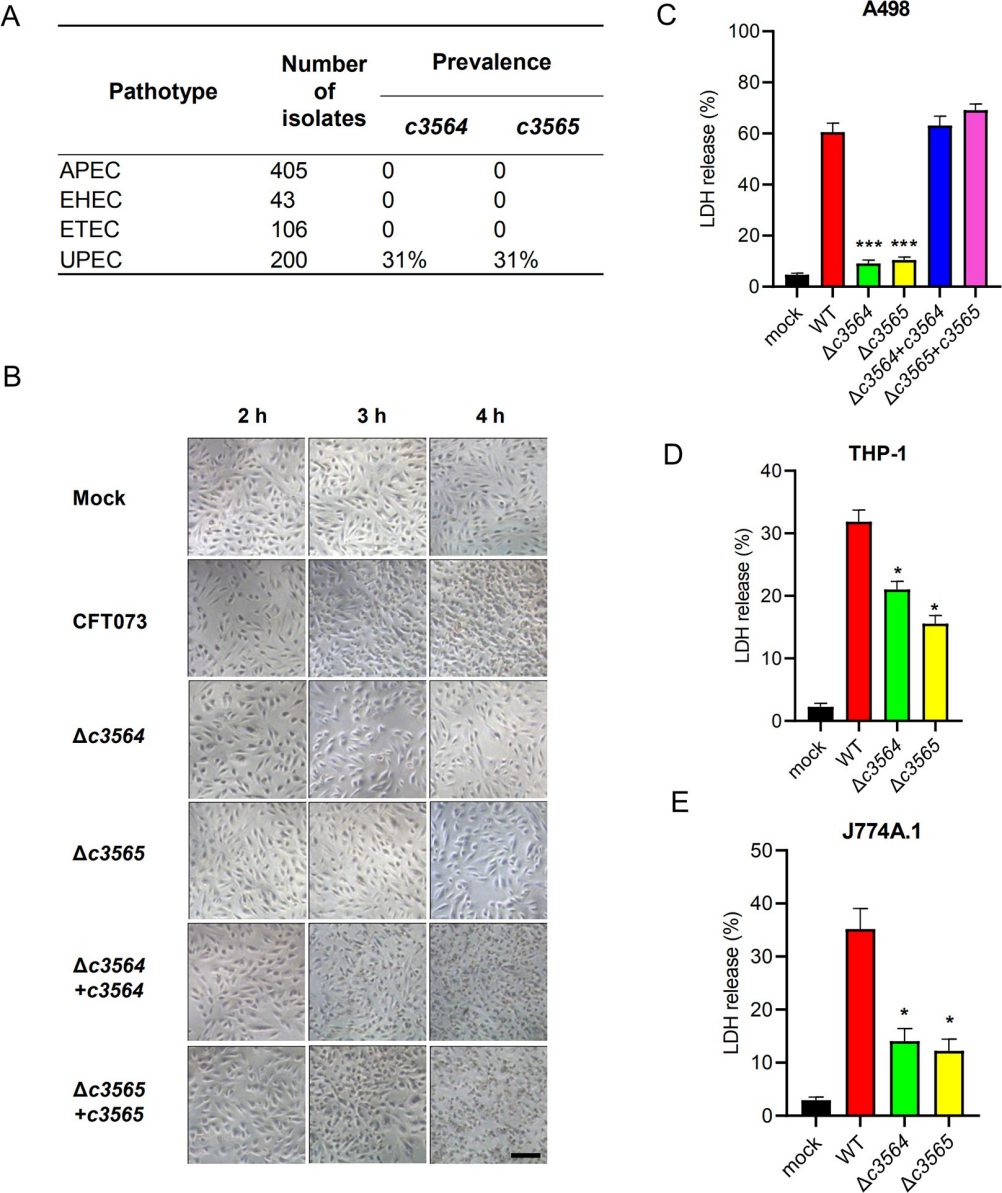
*c3564* and *c3565* promote host cell death during infection in cell culture models. **(A)** Prevalence of *c3564* and *c3565* in various *E*. *coli* pathotypes. A duplex PCR method was used to detect the presence of the *c3564* and *c3565* genes in a laboratory collection of *E*. *coli* isolates. APEC, avian pathogenic *E*. *coli*; EHEC, enterohemorrhagic *E*. *coli*; ETEC, enterotoxigenic *E*. *coli*; UPEC, uropathogenic *E*. *coli*. **(B)** *c3564* and *c3565* affected the morphology of kidney epithelial cells. A498 kidney epithelial cells were infected with CFT073 and its derivatives for the indicated amounts of time and then subjected to phase-contrast microscopy (magnification, 20×). All images are representative of three independent experiments. Scale bar, 50 μm. (**C), (D), and (E)** Cytotoxicity assays on different cell types. A498 kidney epithelial cells (C), THP-1 human macrophages (D), and J774A.1 murine macrophage cells (E) were infected with various bacterial strains at a multiplicity of infection (MOI) of 10 for ~2.5 h, and the cell culture supernatants were then subjected to LDH release measurement. Cytotoxicity (%) was determined by comparing the LDH in culture supernatants to the total cellular LDH (the amount of LDH released upon cell lysis with 0.1% Triton X-100) according to the formula [(experimental − target spontaneous)/(target maximum − target spontaneous)] × 100. The data are the mean ± SD of three replicates from three independent experiments. *, *P <* 0.05; ***, *P <* 0.001 by one-way ANOVA followed by Dunnett’s multiple comparisons test against wild-type CFT073.

To assess the prevalence of *c3564*/*c3565* in pathogenic *E*. *coli* strains, we first performed a BlastN search analysis, and the results demonstrated that *c3564*/*c3565* orthologs can be found in most UPEC genomes available in the NCBI database but not in other pathotypes, such as avian pathogenic *E*. *coli* (APEC), EHEC, and enterotoxigenic *E*. *coli* (ETEC). Duplex PCR detection of *c3564* and *c3565* in a large collection of *E*. *coli* clinical isolates showed that these two genes are highly associated with the uropathogenic pathotype (Fig 2A), implying that *c3564*/*c3565* might be important for UPEC pathogenicity. We then constructed two single-gene deletion mutants of CFT073, Δ*c3564* and Δ*c3565*, and used them to examine whether *c3564* and *c3565* play a role in the interaction between UPEC and host cells. Pronounced cytopathic effects, including cell rounding and detachment, were observed in kidney epithelial cells (A498) after 3 h of infection with wild-type CFT073 (Fig 2B). In contrast, deletion of either *c3564* or *c3565* nearly eliminated the cytopathic effects caused by CFT073, suggesting that *c3564* and *c3565* are involved in damaging host cells. Next, we quantified cell death by using an LDH release assay. Infection with wild-type CFT073 led to substantial A498 cell death, whereas infection with the Δ*c3564* or Δ*c3565* mutant resulted in significantly less cell death (Fig 2C). Introduction of a plasmid-borne *c3564* or *c3565* locus into the corresponding mutant restored the cytopathic effects caused by CFT073 (Fig 2C). Additionally, in two other cell types, the human macrophage-like cell line THP-1 and the mouse macrophage cell line J774A.1, deletion of *c3564* or *c3565* significantly reduced UPEC-induced host cell death (Fig 2D and 2E). Altogether, these data demonstrate that *c3564* and *c3565* promote host cell death during UPEC infection *ex vivo*.

### C3564 and C3565 mediate induction of host cell pyroptosis through controlling hemolysin production

**Fig 3:**
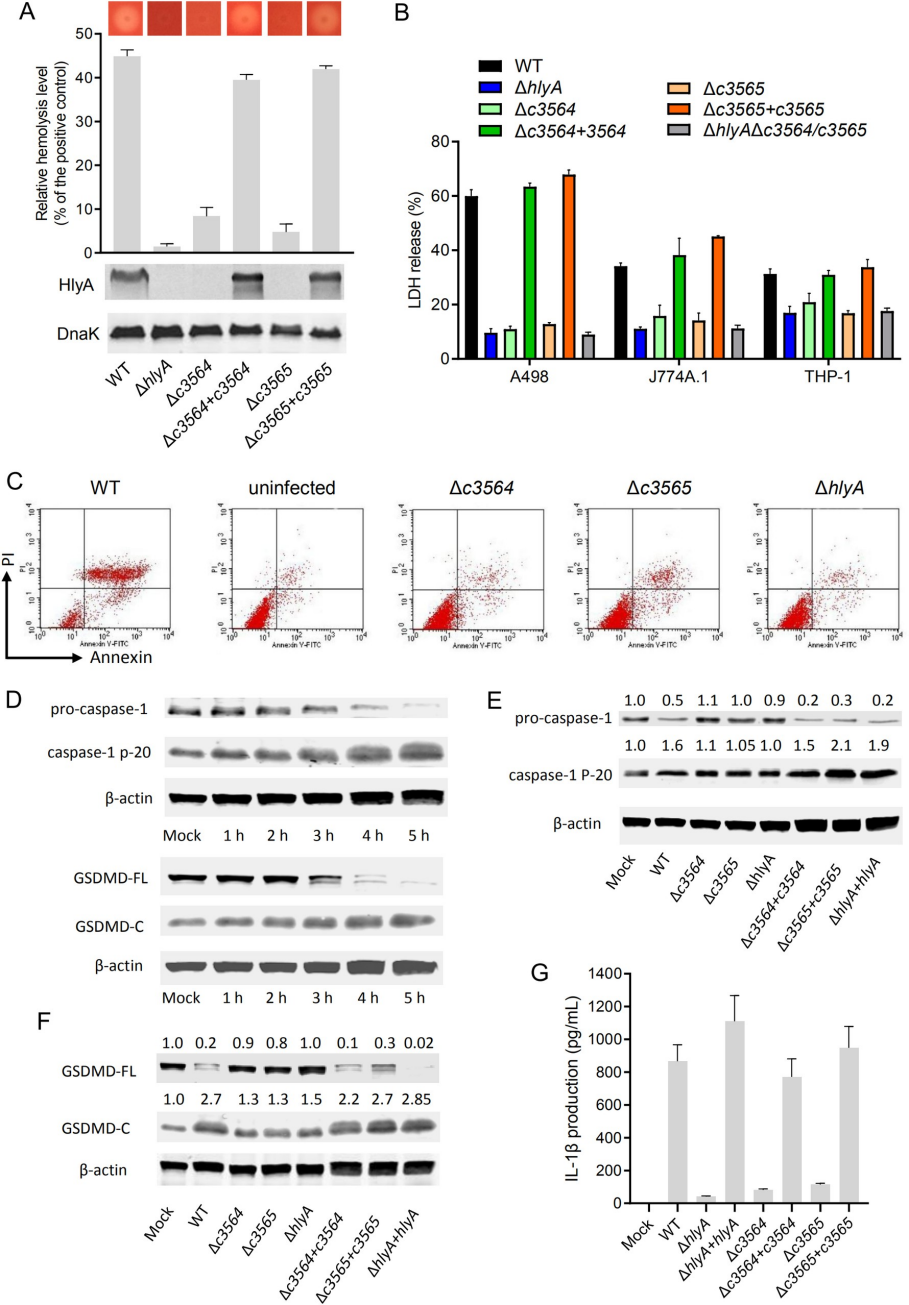
*c3564* and *c3565* promote hemolysin-mediated host cell pyroptosis. **(A)** *c3564* and *c3565* regulate hemolysin production and hemolysis. The dark dot in the center of each image represents a bacterial colony, and the halo around the dark dot represents hemolysis. A wider halo indicates stronger hemolysis. For liquid hemolysis assay, the supernatants of three bacterial culture replicates were incubated with sheep red blood cell solution for 1 h before being subjected to OD_540_ measurement. Triton X-100 (1%), which would lyse the red blood cells completely, was used as a positive control. Percent hemolysis was calculated with reference to the positive control as 100%. For western blot, colonies were picked up by inoculation loops and resuspended in ice-cold PBS, followed by OD_600_ normalization. Bacterial lysates were separated by SDS-PAGE, followed by immunoblotting using antibodies against HlyA and DnaK as a loading control. All images are representative of three independent experiments. **(B)** *c3564-* and *c3565*-mediated hemolysin-induced host cell death in different host cell types. The LDH release assay was done according to Fig 2. *, *P <* 0.05; ***, *P <* 0.001 by one-way ANOVA followed by Dunnett’s multiple comparisons test against wild-type CFT073. **(C)** Analysis of the cell death pathways by Annexin-V/PI staining. A498 cells were infected with various CFT073 strains for 1 h, and then stained with Annexin-V and PI, followed by flow cytometry analysis. Live cells are negative for both stains; early apoptotic cells display high Annexin-V signal in the absence of PI staining; necrotic, pyroptotic and late-apoptotic cells have a high PI signal. Shown are representative images of at least two independent experiments. **(D)** UPEC CFT073 induces the processing of pro-caspase-1 and GSDMD. Cells cultured in 6-well plates were infected with wild-type CFT073 for different lengths of time, and uninfected cells were used as a control. Cell lysates were separated by SDS-PAGE, followed by immunoblotting using antibodies against the indicated proteins including β-actin as a loading control. (**E) and (F)** *c3564* and *c3565* promote hemolysin-mediated processing of pro-caspase-1 (E) and GSDMD (F) during infection of A498 cells. Western blotting was performed as described above. The numbers above the blots indicate the relative expression levels, with that of the mock group set as 1.0, based on densitometry with ImageJ. All blots are representative of ≥2 independent experiments. **(G)** IL-1β production by THP-1 macrophages. Cells were infected with the indicated CFT073 strains (MOI = 10) for 2.5 h. IL-1β release into the supernatants were analyzed by ELISA. Data represent the mean ± SD of three independent experiments.

To elucidate the mechanism by which C3564 and C3565 promote host cell death, RNA-seq analysis was performed to identify genes regulated by C3564 and C3565 during bacterium-cell interactions. Wild-type CFT073 or its mutants associated with A498 cells were collected, and bacterial RNA extraction and transcriptome sequencing were then performed. A 2-fold change was selected as the cutoff. Table 1 lists some of the genes that were most downregulated due to deletion of *c3564* or *c3565*. Notably, the *hlyCABD* genes, responsible for the production and translocation of hemolysin, were downregulated by ~4-fold or more in the Δ*c3564* and Δ*c3565* mutants. Furthermore, immunoblotting and hemolysis assays confirmed that deletion of either *c3564* or *c3565* impaired hemolysin production and subsequent hemolysis, and these phenotypic changes can be properly complemented (Fig 3A). These results indicate that C3564 and C3565 positively regulate hemolysin production.

**Table 1:** Select genes that were differentially expressed in the Δ*c3564* and Δ*c3565* mutants compared to the wild type.

Gene name	log_2_fold change		Product
	Δ*c3565* vs WT	Δ*c3564* vs WT	
*c3565*	NA^a^	-1.02	DNA-binding response regulator
*c3568*	-5.42	-5.17	hypothetical protein
*c3566*	-5.23	-6.79	cytochrome B
*c3567*	-5.19	-5.27	hypothetical protein
*C_RS27520*	-3.48	-3.50	hypothetical protein
*hlyC*	-3.21	-3.06	hemolysin-activating lysine-acyltransferase hlyC
*hlyA*	-2.76	-2.66	RTX toxin hemolysin A
*hlyD*	-2.63	-2.49	HlyD family type I secretion periplasmic adaptor subunit
*c3564*	-2.48	NA^a^	two-component sensor histidine kinase
*lacY*	-2.44	-2.38	MFS transporter
*lacA*	-2.33	-2.37	galactoside O-acetyltransferase
*C_RS16055*	-2.01	-2.27	tRNA-Met
*hlyB*	-1.98	-1.95	alpha-hemolysin translocation ATP-binding protein HlyB
*aroP*	-1.89	-1.99	aromatic amino acid transporter AroP
*c3577*	-1.68	-1.60	transposase

^a^NA, not applicable.

LDH release assays further showed that a *hlyA* mutant produced limited cell death, similar to the Δ*c3564* or Δ*c3565* mutant, and these phenotypes can be properly complemented. Furthermore, deletion of *c3564*/*c3565* in the Δ*hlyA* mutant did not alter cytopathic effects (Fig 3B). Together, these results suggest that C3564/C3565 mediated induction of host cell death through regulating hemolysin. To gain insights into the cell death pathways triggered when *c3564* and *c3565* are present, we infected A498 cells with wild-type CFT073 and its derivative strains, and then used flow cytometry to detect annexin V-FITC/PI staining of the infected cell. Our results showed that within 2 hours post infection (hpi) the majority of cells became double-positive stained (Fig 3C), which is different from necrosis and apoptosis status, suggesting that the infected cells have undergone death pathways like pyroptosis other than apoptosis [29]. And deletion of *c3564*, *c3565*, or *hlyA* reduced annexin V/PI staining, suggesting that these genes are involved in this cell death phenotype (Fig 3C). Further, we found that in A498 kidney epithelial cells infected with wild-type CFT073, pro-caspase-1 was gradually processed into p20, and the production of p20 was increased significantly at 5 hpi; in addition, full-length gasdermin D (GSDMD) was cleaved into GSDMD-N and GSDMD-C (Fig 3D). As GSDMD is the effector molecule that determines cell pyroptosis [30], these results indicate the onset of pyroptosis in A498 cells infected with the wild type. In contrast, deletion of *hlyA* in CFT073 significantly reduced the processing of pro-caspase-1 into caspase-1 (Fig 3E) and the cleavage of GSDMD (Fig 3F), indicating weakened pyroptosis induction by the mutant compared to that induced by the wild type. Thus, *hlyA* is important for CFT073-induced pyroptosis in kidney epithelial cells. When *hlyA* was present, deletion of either *c3564* or *c3565* reduced the occurrence of pyroptosis, and these phenotypic changes due to the gene deletions were able to be properly complemented (Fig 3E and 3F). Caspase-1 activation usually leads to maturation and release of the proinflammatory interleukin 1β (IL-1β), which can recruit neutrophils and macrophages to sites of infection [31], we thus tested whether mutation of *c3564*/*c3565* and *hlyA* influences IL-1β release during UPEC-induced pyroptosis. Our data demonstrate that deletion of *hlyA*, *c3564* or *c3565* significantly reduced IL-1β release into the supernatants (Fig 3G). Furthermore, we also analyzed the roles of HlyA and C3564/C3565 in inducing pyroptosis in other host cell types, including THP-1 and J774A.1 macrophages, and the results showed that Δ*c3564*/*c3565* and Δ*hlyA* mutants presented diminished cleavage of full-length GSDMD, but introduction of *hlyCABD* genes into Δ*c3564*/*c3565* mutant markedly increased cleavage of full-length GSDMD (S1 Fig). Together, these results suggest that C3564/C3565 mediates induction of host cell pyroptosis through regulating bacterial hemolysin production.

### C3564 and C3565 control hemolysin expression by directly regulating *c3566*-*c3568*

**Fig 4:**
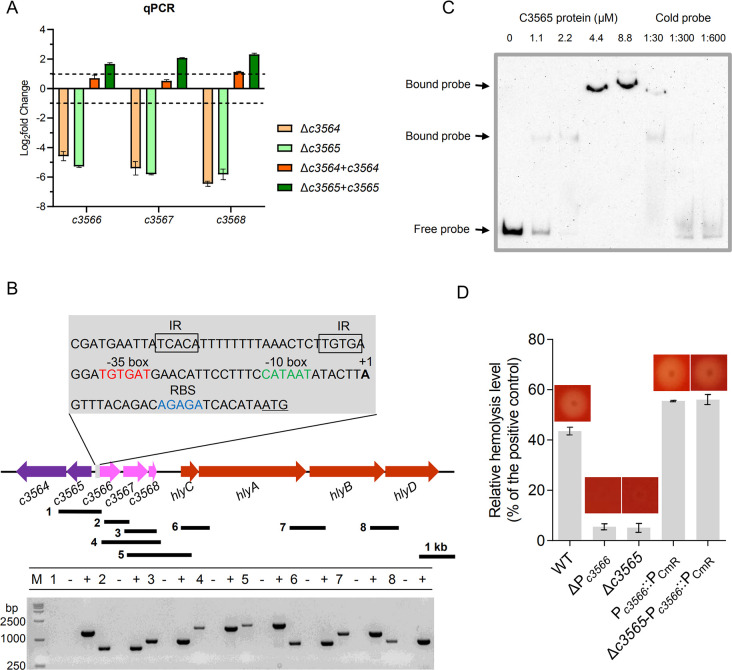
*c3566*-*c3568* transcription and *hlyCABD* transcription are linked and controlled by *c3564* and *c3565*. **(A)** qPCR analysis indicates that *c3564* and *c3565* positively regulate the expression of *c3566*-*c3568* expression. Bacteria were collected by scraping off the plates and immediately stabilized in RNAlater reagent. Total RNA was isolated and reverse transcribed to cDNA. The *rpoB* gene was used as an internal control, and the 2^-ΔΔCt^ method was employed to determine fold-changes in CFT073 variants compared to the wild type. Dashed lines represent a 2-fold change cutoff, and a fold-change ≥2 was considered significant. Values are the mean ± SD of three replicates from 3 independent experiments. **(B)** RT-PCR analysis indicates that the *c3566*-*c3568* operon and *hlyCABD* operon are cotranscribed. RNA and cDNA samples were prepared as described above. The black bars under the gene arrows denote the PCR amplicons generated during testing of cotranscription, and the number in front of each bar corresponds to the lane in the agarose gel. The #1 fragment was used as a negative control reaction, as *c3565* and *c3566* do not cotranscribe. RNA that was not reverse transcribed served as a negative control template, while genomic DNA served as a positive control template. 5’-RACE PCR was used to identify the TSS (+1) of the *c3566*-*c3568* operon, and the -10 and -35 motifs were deduced afterward. Gray box, promoter region of *c3566*; underlined ATG, start codon; blue letters, ribosomal binding site; green letters, -10 box; red letters, -35 box; boxed letters, inverted repeats (IRs) likely bound by the C3565 protein. The genes are drawn to scale. **(C)** EMSA analysis of C3565 protein bound to the *c3566* promoter region. C3565-His_6_ recombinant protein was purified to homogeneity, and the biotinylated *c3566* promoter fragment was used as a probe. Concentrations of the proteins and the ratios of cold probe to the biotinylated probe were indicated above the image. **(D)** Hemolysis is regulated by the *c3566* promoter (P_*c3566*_). Hemolysis assays with liquid blood and blood agar were performed on various CFT073 strains. Strain names represent: WT, wild-type CFT073; ΔP_*c3566*_, *c3566* promoter deletion mutant; Δ*c3565*, *c3565* deletion mutant; P_*c3566*_::P_CmR_, a mutant in which *c3566* promoter is replaced by P_CmR_; Δ*c3565*-P_*c3566*_::P_CmR_, a mutant in which *c3565* is deleted and *c3566* promoter is replaced by P_CmR_. All images are representative of at least two independent experiments, each containing 3 replicates.

Then, we began to explore the mechanism underlying the regulation of hemolysin by C3564/C3565. Among the differentially expressed genes in the Δ*c3564*/wild type and Δ*c3565*/wild type comparisons, *c3566*, *c3567*, and *c3568*, which are immediately downstream of *c3564*/*c3565*, were the genes most downregulated upon knockout (Table 1). Quantitative real-time PCR (qPCR) confirmed that C3564 and C3565 positively regulate the expression of *c3566*, *c3567*, and *c3568* (Fig 4A). Reverse transcription PCR (RT-PCR) results showed that *c3566*, *c3567*, and *c3568* are cotranscribed, and therefore form an operon (Fig 4B). The transcription start site (TSS) of the *c3566*-*c3568* operon was determined by 5’-RACE PCR, and the ribosomal binding site (RBS) as well as the -10 and -35 boxes were deduced using BProm program (http://linux1.softberry.com) (Fig 4B). In an electrophoretic mobility shift assay (EMSA) with a 260 nt DNA fragment containing the promoter region as a probe, we found that the C3565 protein directly binds to the promoter region (Fig 4C). Indeed, a potential RR-binding region containing inverted repeats (TCACA-N_15_-TGTGA) was identified immediately upstream of the -35 box (Fig 4B). Altogether, these data indicate that C3565 directly regulates the *c3566*-*c3568* operon.

We then investigated how C3565 regulates the two adjacent operons, *c3566*-*c3568* and *hlyCABD*. An EMSA demonstrated that the C3565 protein does not associate with a region upstream of the *hlyA* coding sequence (-300 to +1 relative to the *hlyA* start codon) (S2 Fig), suggesting a lack of direct regulation. We therefore hypothesized that C3565 may regulate *hlyCABD* through other *cis-* or *trans*-elements. A series of deletion mutants were generated, including mutants with individual and combined deletions of *c3566*, *c3567*, and *c3568*, as well as various deletions of the intergenic region between *c3568* and *hlyC*, but these genetic modifications did not affect hemolysis (S3 Fig). However, when the promoter P_*c3566*_ (from the -35 box to the TSS) was deleted, hemolysis was lost. Insertion of a constitutively expressed P_CmR_ promoter in front of the *c3566* coding sequence restored hemolysis, and the restoration was independent of *c3565* (Fig 4D). These results imply that *c3566*-*c3568* and *hlyCABD* can be cotranscribed. Indeed, RT-PCR demonstrated that *c3566*-*c3568*-*hlyCABD* can be transcribed as a polycistronic mRNA (Fig 4B). Collectively, these results indicate that C3564 and C3565 control hemolysin expression by directly regulating *c3566*-*c3568-hlyCABD*.

### C3564/C3565 contributes to UPEC resistance to H_2_O_2_ *in vitro* as well as to survival within macrophages through C3566/C3567

**Fig 5:**
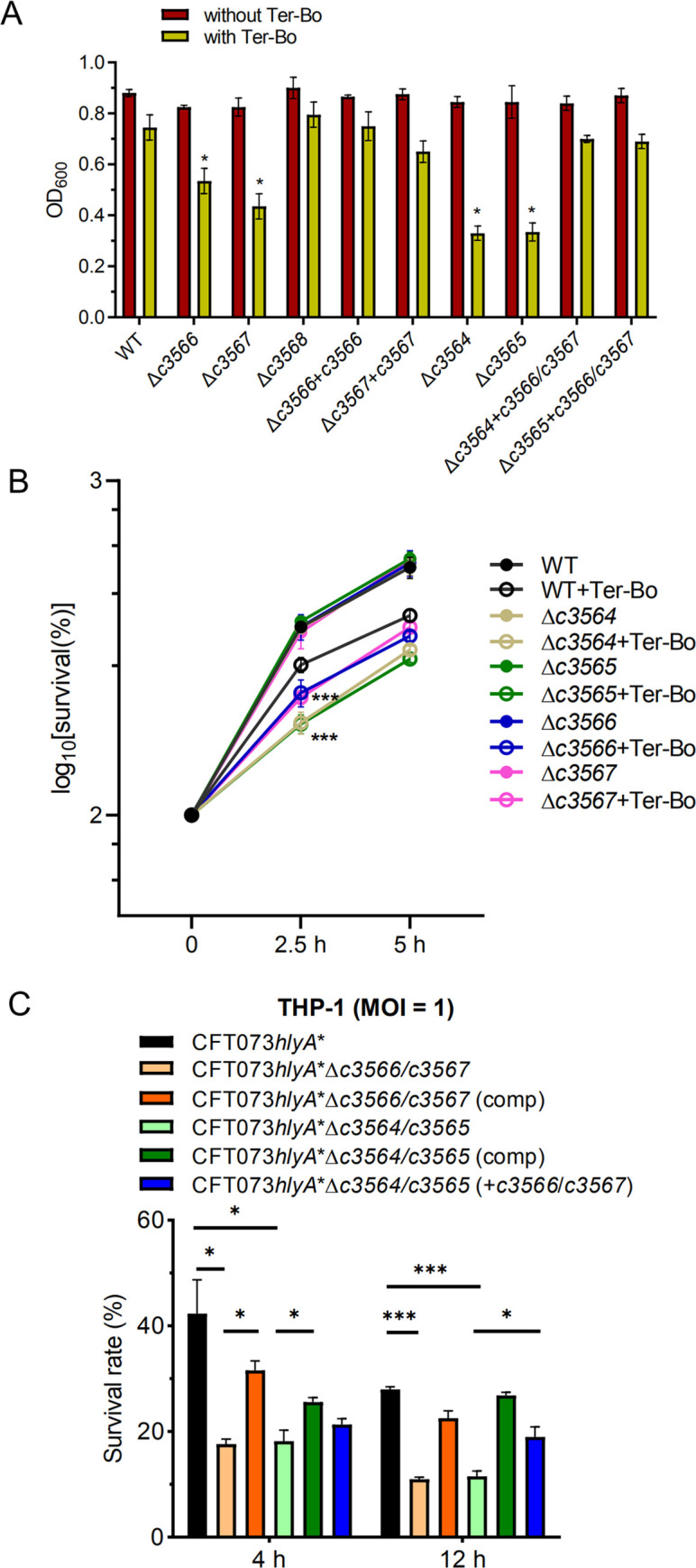
*c3564*/*c3565* and *c3566*-*c3568* mediate oxidative stress resistance and intracellular survival in UPEC. **(A)** Growth inhibition by H_2_O_2_. Tert-butyl hydroperoxide (Ter-Bo) was added to log-phase bacteria grown in minimal medium at a final concentration of 1 mM, and cultures without added Ter-Bo were used as a control. The OD_600_ values were recorded to assess growth. Values are the mean ± SD of three replicates from 3 independent experiments, *, *P* < 0.05 by one-way ANOVA followed by Dunnett’s multiple comparisons test. **(B)** Survival rates of various CFT073 strains under H_2_O_2_ stress. Two equal portions of log-phase culture were taken, with one portion treated with Ter-Bo at a final concentration of 1 mM and the other with PBS as a control. The CFU counts at time-point 0 were set as 100%. Survival rates were determined as [(CFUs at a time-point after stress/CFUs at time-point 0)×100%]. Values are the mean ± SD of three replicates from 3 independent experiments. **(C)** Survival of various CFT073 mutant strains within human macrophages. THP-1 cells were infected with human complement-opsonized CFT073 strains at an MOI of 1 for 45 min at 37°C. A gentamicin protection assay was performed to determine the intracellular bacterial counts at the indicated times. Survival was determined as the mean percentage of the number of bacteria recovered at the indicated times compared to that at 1 h after gentamicin treatment, which was considered 100%. CFT073hlyA*, which carries a partially deleted *hlyA* locus, was used as the parental strain, as it exerts very limited cytopathic effects. Comp indicates the strain carries a complementation plasmid. The data represent the mean ± SD of three replicates from three independent experiments. *, *P* < 0.05; ***, *P* < 0.001 by one-way ANOVA followed by Dunnett’s multiple comparisons test.

Analysis of their amino acid sequences showed that C3567 and C3566 are 27.6% and 23.1% identical to *E*. *coli* MG1655 MsrP and MsrQ, respectively, which encode an Msr system. The periplasmic MsrP catalyzes the reduction of MetSO through its molybdenum-molybdopterin cofactor, while the membrane-bound MsrQ mediates electron supplies for the MetSO reduction likely using the quinone pool of the respiratory chain [32]. Methionine residues in proteins are particularly sensitive to oxidative stressors that can convert methionine into MetSO, which renders proteins dysfunctional [32,33]. Msr system repairs methionine residues damaged by ROS and RCS, including H_2_O_2_ [34]. C3568 encodes a small methionine-rich peptide, and a homolog of C3568 in *Azospira*, MrpX, serves as an oxidative sacrificial sink protein that scavenges HOCl [35,36]. Thus, we hypothesized that the TCS C3564/C3565 contributes to UPEC resistance to H_2_O_2_ through regulating *c3566*-*c3568* expression. In the presence of tert-butyl hydroperoxide (Ter-Bo), compared to the wild-type strain, individual *c3564*, *c3565*, *c3566* and *c3567* deletion mutants exhibited significantly reduced growth (Fig 5A). In contrast, without Ter-Bo, no growth differences were observed between the wild-type and mutant strains. Complementation of *c3566* and *c3567 in trans* restored growth in the presence of Ter-Bo, and constitutive expression of *c3566*-*c3567* in the *c3564* or *c3565* mutant strains restored the growth to the wild-type level (Fig 5A). To corroborate the data above, we also determined the survival rates of the various CFT073 strains based on CFU counts under Ter-Bo stress. In the absence of Ter-Bo, all strains exhibited equivalent growth kinetics; while in the presence of 1 mM Ter-Bo, all individual mutants (Δ*c3564*, Δ*c3565*, Δ*c3566* and Δ*c3567*) presented reduced survival compared to the wild type (Fig 5B). These results indicate that the TCS C3564/C3565 promotes resistance to oxidative stress (Ter-Bo exposure) through regulating *c3566* and *c3567* expression.

Macrophages produce H_2_O_2_ and other oxidants to damage bacterial pathogens [37]. Therefore, we evaluated whether C3564/C3565 and *c3566*-*c3568* are involved in the resistance of UPEC to killing by THP-1 macrophages. As production of HlyA elicits relatively rapid host cell death, thereby preventing long-term incubation of bacteria with host cells (Fig 3B), we generated a *hlyA* partial deletion strain of CFT073 (Δamino acids 564–936, herein named CFT073hlyA*) and its variant strains. To note, compared to the wild type, the CFT073hlyA* induced weaker cell death, to the levels equivalent to that caused by the Δ*hlyA* mutant; and that the CFT073hlyA* and the Δ*hlyA* mutants exhibited similar intracellular survival within macrophages (S4 Fig). Thus, the partial deletion inactivates hemolysin activities yet allows the monitoring of *hlyA* expression (see the subsection below). Deletion of *c3566*/*c3567* or *c3564*/*c3565* from CFT073hlyA* significantly reduced the intracellular survival of bacteria at 4 and 12 h after the addition of gentamycin (Fig 5C). Introduction of plasmid-borne *c3566*/*c3567* or *c3564*/*c3565* into the respective mutants rescued intracellular survival, albeit not to wild-type levels. In addition, constitutive expression of *c3566*/*c3567* in the CFT073hlyA*Δ*c3564* or CFT073hlyA*Δ*c3565* mutant strains significantly increased intracellular survival (Fig 5C). Similarly, *c3566* and *c3567* also contributed to UPEC survival within RAW264.7 murine macrophage cells (S5 Fig). These results indicate that the TCS C3564/C3565 promotes UPEC resistance to killing by macrophages by regulating *c3566* and *c3567* expression.

### C3564/C3565 induces *c3566*-*c3568*-*hlyCABD* expression in response to exogenous H_2_O_2_ and host cell-derived H_2_O_2_

**Fig 6:**
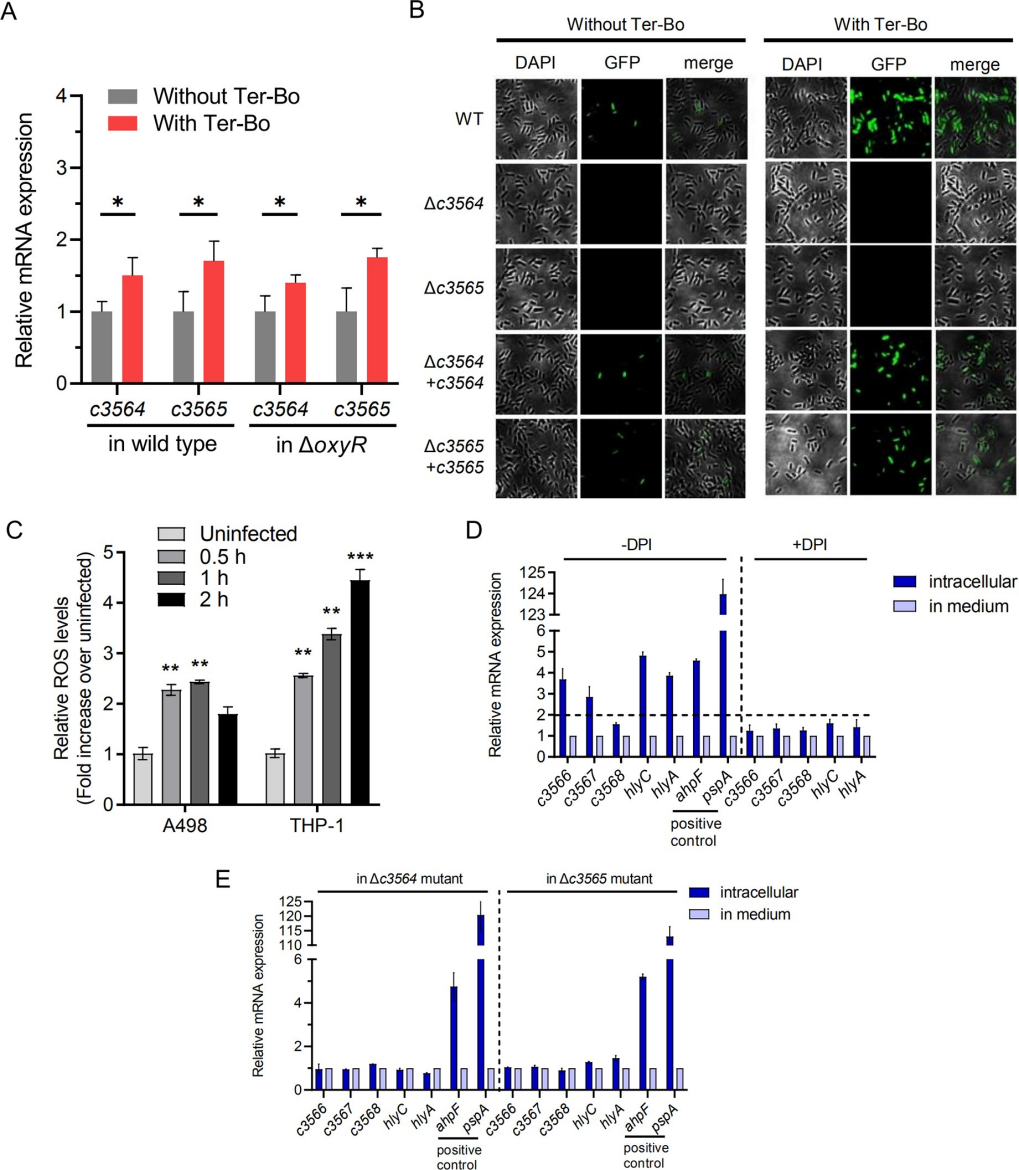
C3564/C3565 activates *c3566*-*c3568*-*hlyCABD* expression in response to *in vitro* and intracellular H_2_O_2_ exposure. **(A)** Stimulation of *c3564* and *c3565* transcription by H_2_O_2_. Bacteria were treated with 1mM Ter-Bo for 10 mins, followed by RNA extraction and qPCR analysis. Values are the mean ± SD of three replicates from 3 independent experiments. *, *P* < 0.05 by Student’s *t* test. **(B)** Regulation of *c3566*-*c3568*-*hlyCABD* expression by C3564/C3565 in response to ROS exposure *in vitro*. GFP activity was monitored in wild-type CFT073 and its mutants, all of which carried a P_*c3566*_-EGFP fusion plasmid. Ter-Bo was added at 1 mM. Representative fluorescence micrographs are shown. **(C)** ROS levels in A498 and THP-1 cells upon UPEC infection. Cells were infected with wild-type CFT073 for various amounts of time and then treated with H_2_DCFDA (20 μM) for 90 min at 37°C. Cellular fluorescence was assessed using a plate reader (excitation/emission at 485 nm/535 nm), and the levels in the uninfected cells were set to 1. Values represent the mean ± SD of 3 biological replicates from 3 independent experiments. **, *P* < 0.01; ***, *P* < 0.001 by one-way ANOVA followed by Dunnett’s multiple comparisons test. **(D)** *c3566*-*c3568*-*hlyCABD* expression induction by intracellular ROS. qPCR was used to examine the transcription levels of *c3566*-*c3568*-*hlyCABD*, and the gene transcription levels in bacteria grown in medium were set as 1. To inhibit the production of ROS, DPI, an inhibitor of NADPH oxidase, was added at 100 μg/mL. The dashed lines represent a fold change cutoff of 2, and a fold change ≥2 was considered significant. **(E)** Regulation of *c3566*-*c3568*-*hlyCABD* intracellular expression by C3564/C3565. The transcription levels of *c3566*-*c3568*-*hlyCABD* in the Δ*c3564* and Δ*c3565* mutants within THP-1 macrophages and in medium were tested by qPCR. The gene transcription levels in bacteria grown in medium were set as 1. The positive control genes *ahpF* and *pspA* have been reported to be induced in UPEC inside macrophages. These expression assays were repeated at least three times in triplicates.

Since C3564/C3565 and its regulated factors C3566/C3567 contribute to UPEC resistance to H_2_O_2,_ we investigated whether the expression of *c3564*/*c3565* and *c3566*-*c3568*-*hlyCABD* responds to the presence of H_2_O_2_ in medium. The qPCR analysis indicated that Ter-Bo significantly stimulated *c3564* and *c3565* expression, although the increase was less than two-fold. Moreover, the stimulation of *c3564*/*c3565* by Ter-Bo was not dependent on OxyR (Fig 6A). Using a P_*c3566*_-EGFP fusion plasmid for transcriptional analysis, we showed that *c3566*-*c3568-hlyCABD* expression in the wild type was significantly higher in the presence of Ter-Bo than in its absence (Fig 6B). Deletion of either *c3564* or *c3565* abolished GFP fluorescence, while introduction of *c3564* or *c3565* into the respective mutant restored GFP fluorescence (Fig 6B), suggesting that *c3564* and *c3565* are required for the induction of *c3566* expression by Ter-Bo.

Then, we suspected that the TCS C3564/C3565 might respond to intracellular H_2_O_2_ during kidney epithelial cell or macrophage infection. First, we found that ROS production within host cells (A498 and THP-1) was significantly induced at 0.5 h after infection with UPEC. As the infection time increased (up to 2 hpi), ROS levels within THP-1 cells increased, and ROS production was induced to a greater degree in THP-1 cells than in A498 cells (Fig 6C). We next used CFT073hlyA* to infect THP-1 macrophages for different lengths of time and at different multiplicities of infection (MOIs). The RNA of intracellular bacteria was isolated, and qPCR was used to assess the transcription levels of *c3566*, *c3567*, *c3568* and *hlyC* and *hlyA*; *ahpF* and *pspA* were used as positive control genes, as the expression of these genes is induced in UPEC within macrophages [38]. As expected, the expression of *ahpF* and *pspA* was dramatically induced in CFT073hlyA* within THP-1 macrophages, and the expression of *c3566*, *c3567*, *c3568*, *hlyC*, and *hlyA* was significantly higher within cells than in medium (Fig 6D). Diphenyleneiodonium chloride (DPI), an NADPH oxidase inhibitor that represses ROS production, impaired the intracellular expression induction, suggesting that intracellular H_2_O_2_ was involved in the induction of these genes (Fig 6D). Furthermore, *c3564* or *c3565* deletion ablated the upregulation of *c3566*, *c3567*, *c3568*, *hlyC*, and *hlyA* within THP-1 cells (Fig 6E), indicating that within macrophages, *c3564* and *c3565* are essential for H_2_O_2_-mediated induction of the *c3566-c3568-hlyCABD* operon. Additionally, these results were reproduced in the mouse macrophage cell line RAW264.7 under interferon-gamma stimulation (S6 Fig), which results in abundant H_2_O_2_ production [39]. Collectively, these data indicate that within host macrophages, *c3566*-*c3568* and *hlyCABD* expression is linked and is regulated by C3564 and C3565.

## Discussion

**Fig 7:**
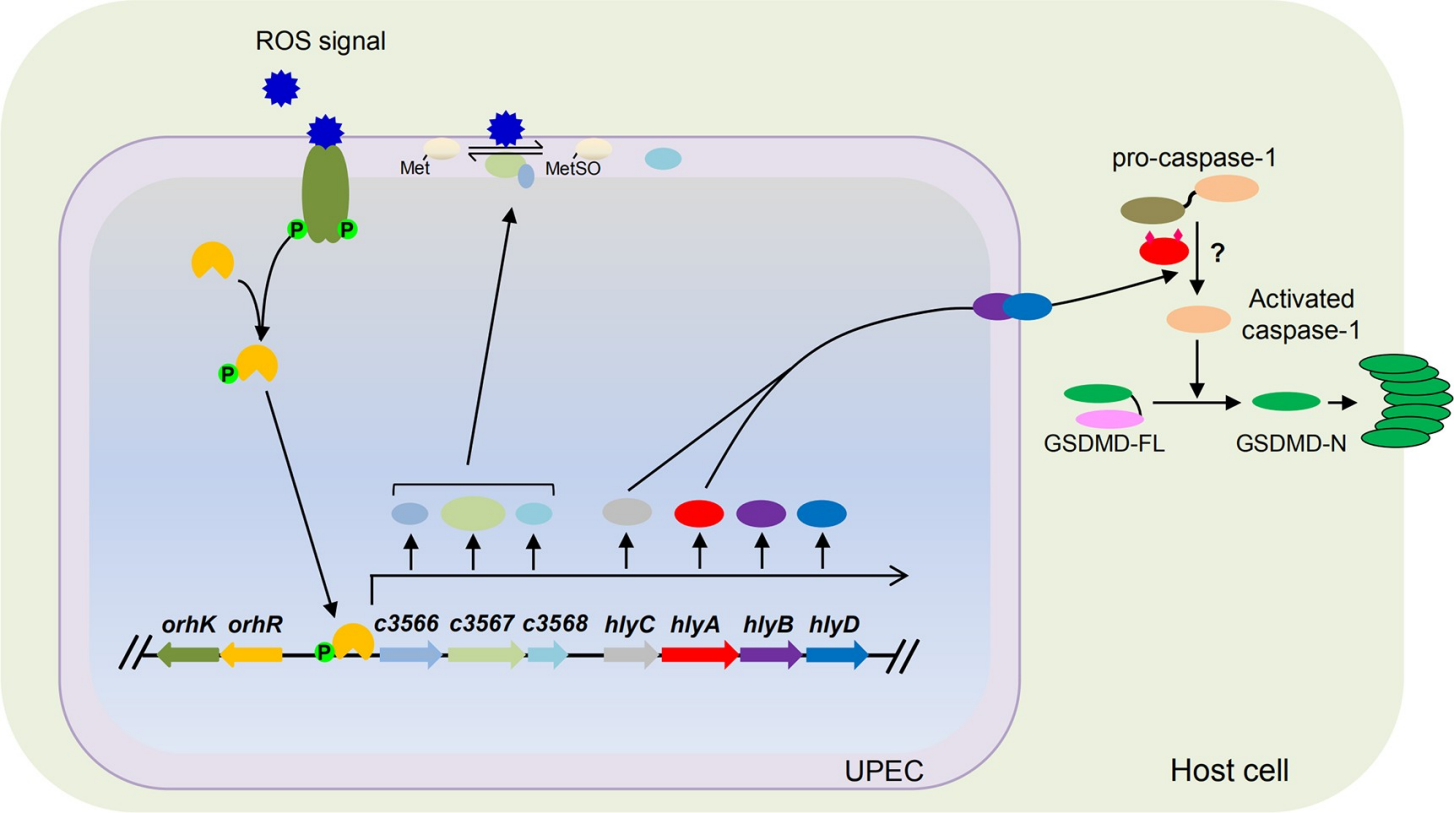
A schematic model of virulence regulation by C3564/C3565 (OrhK/OrhR). We propose that once inside host macrophages, UPEC is able to sense the presence of ROS through the histidine kinase (HK) C3564, which, in turn, transphosphorylates the response regulator (RR) C3565; phosphorylated C3565 is an active form and therefore binds to the promoter region of *c3566* and upregulates *c3566*-*c3568*-*hlyCABD* expression. Upon acylation by HlyC, hemolysin encoded by *hlyA* becomes activated, which is translocated by type I secretion machinery containing HlyB and HlyD. C3566 is thought to be membrane-bound, while C3567 and C3568 are periplasmic, and the Msr system C3566-C3567 functions to repair proteins by converting MetSO to Met, thus providing protection. Meanwhile, increased hemolysin production triggers pro-caspase-1 and GSDMD processing, and the resultant GSDMD-N polymerizes and forms pores on the host cell membrane, eventually causing host cell pyroptosis. The question mark (?) indicates a pathway yet to be discovered.

We found that the TCS C3564/C3565 directly regulates the expression of *c3566-c3568* and its downstream operon *hlyCABD* because of the cotranscription of *c3566-c3568* and *hlyCABD*. In the presence of H_2_O_2_ (particularly during engulfment by macrophages in which H_2_O_2_ is abundantly produced), *c3566*-*c3568* and hemolysin production is greatly induced. UPEC protect themselves from being killed by H_2_O_2_ in immune cells using a putative Msr system encoded by *c3566-c3567*, serving as a “shield”, and produce large amounts of hemolysin, serving as a “sword”, to trigger rapid host cell death, thus minimizing the detrimental effects that macrophages impose on UPEC and facilitating infection by UPEC. Our study therefore depicts a strategy in which UPEC employ a TCS to coordinate oxidative stress resistance and killing of host cells in response to host-derived H_2_O_2_ signals (Fig 7). All these functions are encoded in a gene cluster within a pathogenicity island. Our study thus highlights the crucial role of horizontal gene transfer (HGT) in the evolution of bacterial pathogens. In light of their functions, herein we rename *c3564*/*c3565* as *orhK*/*orhR* (oxidative resistance and hemolysis kinase/regulator).

Based on their homology with the YedVW TCS-regulated MsrPQ, C3566 and C3567 encode the membrane-bound and periplasmic components, respectively, of an Msr system. In contrast to MsrPQ, which protects *E*. *coli* from RCS, the C3566/C3567 system protects UPEC from ROS (Fig 5). MsrA and MsrB in *E*. *coli* reduce methionine-*S*-sulfoxide and methionine-*R*-sulfoxide, respectively, and function mainly within the cytoplasm [40]. The C3566/C3567 system, however, may serve to repair periplasmic proteins and maintain envelope integrity under conditions of oxidative stress, as the substrates of MsrPQ are periplasmic proteins (the chaperone SurA and lipoprotein Pal) [32]. The identities of the C3566/C3567 substrates are currently under investigation in our laboratory. Therefore, our study identifies a potentially new antioxidative system in UPEC.

The role of *c3566-c3568* obviously goes beyond antioxidation; the promoter P*c3566* is necessary for the transcriptional linkage of *c3566-c3568* and *hlyCABD* (Fig 4), and there is presumably no strong terminator after *c3568* to prematurely stop transcription. Nhu et al. demonstrated that insertion of a transposon that carries a KmR gene oriented towards *hlyCABD* within *c3566-c3568* can elevate the expression of *hlyCABD* [24]. These results support our finding that *c3566-c3568* and the *hlyCABD* operon are cotranscribed. Notably, UPEC strains, such as J96 and 536, contain two functional copies of *hlyCABD*, *hly*_I_ and *hly*_II_. A great majority of *hlyA*-positive UPEC isolates in the NCBI database carry the *hly*_I_ copy alone, including CFT073, UTI89, and S65EC [41]. Although the coding regions of *hlyCABD*_I_ and *hlyCABD*_II_ are nearly identical, their promoter regions are highly dissimilar, thus leading to different expression levels [41,42]. A genotyping analysis of our laboratory collection of 200 UPEC isolates showed that 1) 32% (64/200) of the UPEC isolates possess *hlyA*_I_ operon and display hemolytic activities; 2) ~96.7% (62/64) of the 64 *hlyA*_I_-positive isolates carry *orhK*/*orhR*, whereas only 2 *hlyA*_I_-positive isolates do not carry *orhK*/*orhR*; and 3) all of the 62 *orhK*/*orhR*-positive isolates possess *hlyA*_I_ operon. Similarly, using a well-define set of 246 UPEC isolates previously reported [43] we found that 1) 28.9% (71/246) of the UPEC isolates possess *hlyA*_I_ gene; 2) ~95.8% (68/71) of the 71 *hlyA*_I_-positive isolates carry *orhK*/*orhR*; and 3) ~97.1% (68/70) of the 70 *orhK*/*orhR*-positive isolates possess *hlyA*_I_ operon (S3 Table). Altogether, these data suggest that the presence of *orhK*/*orhR* and *hlyA* operons is highly correlated. Since hemolysin is associated with more severe disease outcomes [44], the linkage of *c3566*-*c3568* with hemolysin and their regulation by OrhK/OrhR could constitute an important mechanism underpinning severe disease caused by hemolysin-positive UPEC.

It has long been thought that pyroptosis is a form of caspase-1-mediated proinflammatory cell death and that caspase-1 processing sufficiently indicates pyroptosis [45]. Recently, it was found that caspase-11/4/5 can also induce pyroptosis [30,46] and that pyroptosis is not necessarily linked to the release of mature IL-1β [47]. Regardless of the caspase activation and cytokine release, increasing lines of evidence show that the real effectors for pyroptosis are the gasdermin-family proteins, primarily GSDMD; upon cleavage, GSDMD-N travels to the membrane and forms pores, allowing the release of cytokines and eventually leading to membrane rupture [48]. UPEC can induce caspase-1/4-dependent inflammatory cell death in bladder epithelial cells [20]. In this study, we further demonstrated that hemolysin induces caspase-1 processing and GSDMD cleavage in both kidney epithelial cells and macrophages, clearly indicating pyroptotic cell death (Fig 3). Hemolysin plays divergent roles in different types of cells from different hosts [44]. This diversity could be at least partially due to different recognition mechanisms of the host cells; some of the various effects exerted by hemolysin are direct, and others are indirect [44].

Pyroptosis is a defense mechanism by which the host eliminates intracellular pathogens [49]. However, this mechanism can be hijacked by pathogens, and excessive pyroptosis can lead to excessive cytokine levels, cell death, and, eventually, tissue damage/scarring and bacterial dissemination into deeper tissue [49]. Our results show that hemolysin apparently induces pyroptosis in kidney cells and macrophages (Fig 3). Accordingly, reports have indicated that expression of hemolysin is associated with more extensive tissue damage within the urinary tract and more severe clinical outcomes [50]. However, previous work has also elucidated that hemolysin overexpression during acute bladder infection induces rapid and extensive exfoliation and reduces bladder bacterial burdens [51]. Therefore, the balance and timing of hemolysin expression are important in determining disease outcomes. The OrhK/OrhR TCS, together with other reported regulators such as CpxA/CpxR [20] and FNR [21], could coordinate to fine-tune hemolysin expression to enhance UPEC fitness during UTI.

Our results showed that OrhK/OrhR acts independently of CpxR in our experimental settings, because similar to the Δ*orhK*/*orhR* vs. WT comparison, deletion of *orhK*/*orhR* in the Δ*cpxR* mutant still led to dramatic decrease in *c3566-c3568*-*hlyCABD* expression (S7 Fig). Additionally, we found that deletion of *oxyR* in the wild type did not affect *c3566-c3568*-*hlyCABD* expression, and OxyR is dispensable for the regulation of *c3566-c3568*-*hlyCABD* by OrhK/OrhR. Likewise, in the presence or absence of *orhK*/*orhR*, OxyR serves as a positive regulator of a target gene *katG* (S8 Fig). Hence, OrhK/OrhR and OxyR represent separate pathways in responding to H_2_O_2_ stress.

In summary, our findings suggest a model in which OrhK/OrhR activates and coordinates the expression of oxidative stress resistance proteins and hemolysin in response to H_2_O_2_ within macrophages (Fig 7). This regulatory circuit protects UPEC from intracellular oxidative stress and simultaneously promotes hemolysin-mediated pyroptotic cell death of host cells, potentially leading to host tissue damage. Our study provides novel insights into bacterial virulence strategies and suggests OrhK/OrhR as a potential antimicrobial dual target to relieve tissue damage during UTI.

## Materials and methods

### Genetic engineering and construction of recombinant plasmids

Strains and plasmids used in this study are listed in S1 Table. DNA amplification, ligation, and electroporation were performed according to standard protocols. All oligonucleotides purchased from Integrated DNA Technologies (Iowa) and Genewiz (Suzhou, China) are listed in S2 Table. The various constructs were confirmed by PCR and DNA sequencing (Core Facility, Iowa State University).

Gene deletions in CFT073 were performed using the Lambda Red recombination system described by Datsenko and Wanner [52]. For complementation, the coding regions were amplified from CFT073 genomic DNA and cloned into a modified plasmid (pGEN-P_CmR_) in which the promoter was replaced with the promoter of the chloramphenicol resistance gene from pKD3. For construction of expression plasmids, fragments of the target genes were obtained using the primers in S2 Table and subsequently cloned into pET21a (Novagen) or pGEX-6P-3 (GE Healthcare). P_*c3566*_ (from the -35 box to the TSS) was deleted, and the expression of *orhK*/*orhR* was not affected, as determined by qPCR.

### In silico and PCR genotyping

The nucleotide sequences of *orhK*, *orhR*, *c3566*, *c3567*, *c3568* and their orthologs in *E*. *coli* were aligned using the ClustalW2 program. Primers were designed from a conserved region on the basis of G/C content, annealing temperatures, and amplicon sizes. Multiplex PCR was carried out according to the methods of Johnson et al [53].

To test the presence of *orhK* (C_RS16920), *orhR* (C_RS16925), and *hlyA* (C_RS16950) in the genomes of a set of UPEC isolates [43], the ssearch36 function of FASTA package was used. If the program outputs a sequence with identity ≥95% and coverage ≥300 bp compared to the reference gene sequence, that gene is considered to be present in the genome.

### Protein expression and purification

To achieve high expression levels of the target proteins (the cytoplasmic domains of OrhK-GST and His_6_-OrhR), recombinant plasmids were transformed into competent *E*. *coli* BL21 cells, and their expression was induced by IPTG under optimal growth conditions. The cells were harvested by centrifugation, washed once with lysis buffer, and stored at -80°C until use. The frozen cells were resuspended in lysis buffer containing phenylmethylsulfonyl fluoride and lysozyme and further lysed by sonication. After centrifugation, the supernatant was extracted with a nickel-nitrilotriacetic acid-agarose suspension (Qiagen) or a Pierce GST Spin Purification Kit (Thermo) as described by the manufacturer. Proteins were eluted, recovered and dialyzed against storage buffer [54]. Finally, the protein concentrations were determined with a protein assay kit (Bio-Rad), and the purity was assessed by SDS-PAGE.

### Autophosphorylation and transphosphorylation

Autophosphorylation and transphosphorylation assays were performed as previously described by Yamamoto et al [54]. For autophosphorylation, purified OrhK-GST was diluted with kinase buffer, and the phosphorylation reaction was initiated by adding ATP. The reaction was carried out at 37°C for various amounts of time and terminated by adding an equal volume of SDS-PAGE sample loading buffer. After SDS-PAGE separation, the proteins in the gel were transferred onto a nitrocellulose membrane and detected using a pIMAGO-biotin Phosphoprotein Detection Kit (TYMORA). For transphosphorylation, the phosphorylated form of OrhK prepared as mentioned above was mixed with a mixture of His_6_-OrhR and excess ATP on ice, and the resulting solution was incubated at 37°C for various amounts of time. Reaction termination and subsequent detection of transphosphorylated proteins were performed exactly as mentioned above.

### Cell morphology assay

A498 cells were infected with various bacterial strains in 24-well plates at an MOI of 10 at 37°C, and uninfected cells were used as controls. At 2, 3, and 4 hpi, cell monolayers were visualized with an Axiovert 40C inverted optical microscope (Carl Zeiss), and images were captured with an EOS 1000D Canon camera at 20× magnification.

### LDH cytotoxicity assay

A498, J774A.1, and THP-1 cells were seeded onto 96-well plates until ~80% confluency was reached. The cells were infected with various bacterial strains at an MOI of ~10. Cell culture supernatants were collected at 2.5 hpi and subjected to LDH release measurement using an LDH Cytotoxicity Assay Kit (C20301). Cytotoxicity (%) was determined by comparing the LDH in culture supernatants to the total cellular LDH (the amount of LDH released upon cell lysis with 0.1% Triton X-100) according to the formula [(experimental − target spontaneous)/(target maximum − target spontaneous)] × 100 [55,56].

### Annexin-V/PI staining and flow cytometry analysis

This experiment was carried out as previously described [29]. Briefly, cells were trypsinized, washed with cold PBS, and then resuspended in the binding buffer [10 mM HEPES (pH 7.4), 140 mM NaCl and 2.5 mM CaCl_2_]. One hundred microliter of the cell suspension were transferred into a new tube and stained with 5 μL FITC-Annexin V and 10 μL propidium iodine for 15 min in dark at room temperature. After incubation, 400 μL of binding buffer was added to the solution and further analyzed by FACScallibur flow cytometer (BD Biosciences).

### Hemolysis assays

Bacterial strains were streaked on sheep blood agar plates (BD Biosciences, NJ), and the plates were incubated at 37°C for 12 h. Colonies of similar sizes were photographed digitally with a stereomicroscope (Olympus) under the same lighting and magnification conditions [20].

Hemolysis assay in liquid was performed as previously described [57], but with minor modifications. Bacteria were cultured in TSB medium at 37°C for 24 h, and then culture supernatants were collected by centrifugation and filtering after OD_600_ normalization. Sheep red blood cells (Luqiao, China) were prepared by washing 3× with PBS. Culture supernatants (245 μl) were combined with 5 μl of washed sheep blood in the presence of 10 mM CaCl_2_ and incubated for 1 h at 37°C statically. TSB was used in place of culture supernatant as a negative control, and 1% Triton X-100 was used as a positive control. After incubation, samples were centrifuged, and hemoglobin release was determined by measuring the optical density at 540 nm (OD_540_). Percent hemolysis was calculated by setting the positive control as 100% hemolysis.

### Transcriptomics by RNA sequencing (RNA-seq)

RNA-seq analysis was performed using a standard protocol, with minor modifications [58]. Briefly, RNA extraction was carried out using a TRIzol (Thermo) method and was followed by determination of the RNA quality and concentration with an Agilent 2100 Bioanalyzer (Agilent Technologies) and a NanoDrop instrument (Thermo Fisher Scientific Inc.), respectively. One microgram of high-quality RNA (A260/A280 ratio ˃ 2.0 and RNA integrity number (RIN) ˃ 7.0) was used for construction of each NextGen sequencing library. Ribosomal RNA was removed from total RNA using a Ribo-Zero rRNA Removal Kit for Bacteria (Illumina). The libraries were constructed according to the manufacturer’s protocol (NEBNext Ultra Directional RNA Library Prep Kit for Illumina). Sequencing of the libraries was performed using a 2x150 paired-end (PE) configuration on an Illumina HiSeq platform according to the manufacturer’s instructions (Illumina, CA); image analysis and base calling were conducted by HiSeq Control Software (HCS) + Off-Line Basecaller (OLB) + GAPipeline-1.6 (Illumina) on the HiSeq instrument. The sequences were processed and analyzed, and the raw reads were assessed with FastQC and further processed with Cutadapt (version 1.9.1). The clean reads were then aligned to the UPEC CFT073 genome (GenBank accession: NC_004431.1) using Bowtie 2 (version 2.1.9, standard options). The reads were counted using HTSeq (version 0.6.1). Differential gene expression analysis was then performed using DESeq2 (version 1.6.3) with R version 3.3.2 following a standard workflow [58]. All genes with a |log_2_(fold change)|>1 and a Benjamini-Hochberg adjusted *P* value (*q* value) < 0.05 were considered differentially expressed.

### RT-PCR and qPCR

UPEC CFT073 and its derivatives were treated with RNAprotect Bacterial Reagent (Qiagen, CA), and RNA was extracted using an RNeasy Mini Kit (Qiagen) with one-hour in-tube DNase digestion (Qiagen) to remove possible DNA contamination according to the manufacturer’s instructions. One microgram of total RNA was reverse transcribed in triplicate using random hexamers and SuperScript IV reverse transcriptase (Invitrogen). For the cotranscription test by RT-PCR, primer pairs were designed to span adjacent genes. RNA that was not reverse transcribed served as a negative control, while genomic DNA served as a positive control [27].

For qPCR analysis, cDNA was used as a template for SYBR Green-based qPCR using TB Green Premix Ex Taq II Reagent (Clontech) and an ABI Quant 5 thermocycler (Applied Biosystems). Melting curve analyses were performed after each reaction to ensure amplification specificity. Fold changes in transcript levels were calculated using the 2^−ΔΔCt^ method [59], and the levels were normalized according to *rpoB* expression. Differences between groups were analyzed by Student’s *t* test.

### EMSAs

To study the binding of OrhR to DNA probes, EMSAs were performed using a LightShift Chemiluminescent EMSA Kit (Thermo) [60]. The OrhR-His_6_ fusion protein was purified to homogeneity using Ni-NTA Spin Columns (Qiagen) and dialyzed against the binding buffer. The DNA probes were PCR amplified using biotinylated primers and gel purified using a Qiagen MinElute Gel Extraction Kit (Qiagen). EMSAs were performed by adding increasing amounts of purified OrhR-His_6_ fusion protein to the labeled probe (2 fmol) in the binding buffer (10 mM Tris (pH 7.5), 1 mM EDTA, 1 mM dithiothreitol, 90 mM KCl, 10 mM MgCl_2_, 10 mM acetyl phosphate, 50 ng/μL poly (dI-dC), 1 μg/mL bovine serum albumin, 5% glycerol) for a 30-min incubation at room temperature. Unlabeled cold probes were added at the ratios indicated. The reaction mixtures were then subjected to electrophoresis on a 6% polyacrylamide gel in 0.5× TBE buffer (44.5 mM Tris, 44.5 mM boric acid, 1 mM EDTA, pH 8.0) at 100 V for 120 mins. The gel was transferred to a nylon membrane and detected according to the kit instructions, and the image was visualized and captured under a ProteinSimple FluorChem imager.

### Mapping of TSSs by 5’-RACE

Total RNA was isolated from UPEC strains as described above. TSS mapping was performed using an ExactSTAR Eukaryotic mRNA 5’- and 3’-RACE Kit (Epicentre Biotechnologies) as described in the manufacturer’s guidelines, except that the first two steps prior to the treatment of RNA with tobacco acid pyrophosphatase (TAP) were omitted [61]. The sequences for the primers used can be found in S2 Table.

### Growth inhibition by H_2_O_2_ (Ter-Bo)

Overnight cultures of CFT073 and its mutants were diluted 1:100 in M9 minimal medium (containing 0.4% glucose) and allowed to grow until they reached an optical density at 600 nm (OD_600_) of ~0.2. To test bacterial resistance to oxidative stress, Ter-Bo was added to the cultures at a final concentration of 1 mM, and cultures without Ter-Bo were used as controls. After incubation for 3 h at 37°C, the H_2_O_2_ resistance was measured by determining the OD_600_ [62]. For survival rate measurement, the OD_600_ values were first normalized, and then two equal portions were taken, with one portion treated with Ter-Bo at a final concentration of 1 mM and the other with PBS as a control. These cultures were collected at various time points, and spotted onto LB agar to determine the CFU counts. The CFU counts at time-point 0 were set as 100%. Survival rates were determined as [(CFUs at a time-point after stress/CFUs at time-point 0)×100%].

### Bacterial intracellular survival assay

Macrophages were cultured in 24-well plates and treated with 100 nM phorbol myristate acetate (PMA) for 48 h for differentiation. The cells were infected with human complement-opsonized CFT073 strains at an MOI of 1 for 45 min at 37°C. After infection, the cells were washed three times with PBS and incubated in medium containing 100 μg/mL gentamicin for 1 h to kill extracellular bacteria. The medium was then replaced with maintenance medium containing 10 μg/mL gentamicin for the indicated amounts of time. Afterward, the monolayers were washed with PBS and lysed with 1% Triton X-100, and the released intracellular bacteria were serially diluted and plated on LB agar for enumeration. Survival was determined as the mean percentage of the number of bacteria recovered at the indicated times compared to that at 1 h after gentamicin treatment, which was considered 100% [63,64].

### RNA isolation from intracellular UPEC

Bacterial infection and RNA isolation from intracellular bacteria were conducted according to previously reported methods [65]. THP-1 cells (~10^6^ cells) were seeded in 6-well plates, stimulated with PMA, and then challenged with human complement-opsonized CFT073 strains at an MOI of 10 for 45 min at 37°C. In parallel, bacteria were incubated in cell-free 6-well plates as the control group. After infection, the cells were washed three times with PBS and incubated in medium containing 100 μg/mL gentamicin (DPI was added at this step when needed) for 1 h to kill the extracellular bacteria. The infected macrophages were then lysed in ice-cold RNA stabilization solution (0.2% SDS, 19% ethanol, 1% acidic phenol in water) and incubated on ice for 30 min to prevent RNA degradation. The lysates containing intracellular UPEC were collected and centrifuged, and RNA was extracted from the bacterial pellets with TRIzol.

### Immunoblotting

Cells were collected and lysed in 5× SDS loading buffer to obtain protein samples. A standard SDS-PAGE protocol was used to separate the proteins. The separated proteins were transferred to PVDF membranes and detected with an anti-GSDMD or anti-Caspase-1 rabbit monoclonal primary antibody and a horseradish peroxidase-conjugated secondary antibody as previously described [66]. GAPDH was used as a loading control. The band intensities were quantified using ImageJ densitometry analysis.

### Enzyme-linked immunosorbent assay (ELISA)

PMA-treated THP-1 macrophages were cultured in 24-well plates and infected with human complement-opsonized CFT073 strains at an MOI of 10 for 2.5 h at 37°C. After infection, cell culture supernatants were collected and subjected to IL-1β measurement using the Human IL-1β ELISA Kit (Beyotime).

### ROS measurement

ROS production in macrophages and kidney epithelial cells was measured as previously described [67], with minor modifications. In 6-well plates, cells were seeded and infected with wild-type CFT073 for the indicated amounts of time. Then, the cells were washed two times with medium and subjected to treatment with 2’,7’-dichlorodihydrofluorescein diacetate (H_2_DCFDA, 20 μM) for 90 min at 37°C. The cells were collected and resuspended in PBS, and the resultant cell suspensions were transferred into 96-well black plates for fluorescence measurement in a plate reader (SpectraMax M3) at an excitation wavelength of 485 nm and an emission wavelength of 535 nm. The mean intensities of uninfected cells were used as controls. The fold changes in fluorescence were calculated by dividing the mean intensity values of the infected groups by those of the uninfected controls.

### Immunostaining and confocal microscopy

Cells (A498 or RAW264.7) were cultured on cell culture dishes, infected with bacterial strains for different time periods, washed, fixed for 15 min with 4% paraformaldehyde in PBS, permeabilized for 20 min in 0.1% Triton X-100 in PBS and blocked using 5% BSA for 1 h. Then, the cells were stained with the indicated corresponding primary antibodies and incubated with fluorescence-conjugated goat anti-mouse IgG (Invitrogen). The nuclei were counterstained with DAPI (Cell Signaling). The slides were mounted using Fluorescence Antifade Mountant (Molecular Probes). Images were captured at room temperature using a confocal microscope (Olympus FluoView FV1000 Confocal System) with a 63× oil immersion objective and Olympus FluoView software (Olympus). The confocal images shown are representative of three independent experiments.

### Statistical analysis

One-way ANOVA (followed by Dunnett’s multiple comparisons test) was used to analyze differences between various mutants and the wild-type strain (GraphPad 9.0, Prism). Student’s *t* test was used for all other binary comparisons. A *P* value < 0.05 was considered to indicate statistical significance.

## Supporting information

S1 Table Strains and plasmids used in this study. (DOC)

S2 Table Oligonucleotides used in this study. (DOCX)

S3 Table Presence of *orhK*, *orhR*, and *hlyA* genes in the genomes of a well-define set of UPEC isolates. (XLSX)

S1 Fig *c3564* and *c3565* promote hemolysin-mediated pyroptosis in human THP-1 and murine J774A.1 macrophages. (A) *c3564*/*c3565* promotion of hemolysin-mediated processing of GSDMD during infection of murine J774A.1 macrophages. LPS plus nigericin, a positive control, were used to induce pyroptosis. (B) *c3564* and *c3565* promote hemolysin-mediated processing of GSDMD during infection of human THP-1 macrophages. Nigericin, a positive control, was used to induce pyroptosis. All blots are representative of ≥2 independent experiments.
(TIF)

S2 Fig C3565 protein does not bind to the *hlyCABD* promoter region as revealed by EMSA. C3565-His_6_ recombinant protein was purified to homogeneity, and the *hlyCABD* promoter probe was PCR amplified and gel purified. Protein concentrations were indicated above the image. DNA probe was stained by SYBR Gold nucleic acid stain.
(TIF)

S3 Fig Hemolysis induced by various CFT073 strains on sheep blood agar. Hemolysis induced by various CFT073 strains on sheep blood agar. Wild-type CFT073 and its deletion mutants are numbered, and the corresponding deleted regions are represented by arrows with crosses in the middle. The dark dot in the center of each image represents a bacterial colony, and the halo around the dark dot represents hemolysis. A wider halo indicates stronger hemolysis. The numbers, 350, 250, and 100, denote the distances between the endpoint of that deletion and the *hlyC* start codon.
(TIF)

S4 Fig Phenotypic comparison of the CFT073hlyA* and the Δ*hlyA* mutants. (A) Cytotoxicity assay. THP-1 human macrophages and J774A.1 murine macrophage cells were infected with various bacterial strains at a multiplicity of infection (MOI) of 10 for ~2.5 h, and the cell culture supernatants were then subjected to LDH release measurement. Cytotoxicity (%) was determined by comparing the LDH in culture supernatants to the total cellular LDH (the amount of LDH released upon cell lysis with 0.1% Triton X-100) according to the formula [(experimental − target spontaneous)/(target maximum − target spontaneous)] × 100. The data are the mean ± SD from three independent experiments. (B) Bacterial survival within macrophages. THP-1 cells were infected with human complement-opsonized CFT073 strains at an MOI of 1 for 45 min at 37°C. A gentamicin protection assay was performed to determine the intracellular bacterial counts at the indicated times. The data represent the mean ± SD from three independent experiments.
(TIF)

S5 Fig C3566 and C3567 mediate survival of CFT073 within murine RAW264.7 macrophages. RAW264.7 cells were infected with CFT073 strains at an MOI of 1 for 45 min at 37°C. A gentamicin protection assay was performed to determine the intracellular bacterial counts at the indicated times. Survival was determined as the mean percentage of the number of bacteria recovered at the indicated times compared to that at 1 h after gentamicin treatment, which was considered 100%. The data represent the mean ±SD of three independent experiments. *, *P* < 0.05 by one-way ANOVA followed by Dunnett’s multiple comparisons test.
(TIF)

S6 Fig *c3566*-*c3568*-*hlyA* expression is regulated by *c3564* and *c3565* during UPEC intracellular infection of murine RAW264.7 macrophages (primed with interferon-gamma). (A, B, C, and D) qPCR was used to examine the transcription levels of *c3566* (A), *c3567* (B), *c3568* (C), and *hlyA* (D), and the gene transcription levels in bacteria grown in medium were set as 1. WT, wild-type CFT073. (E) Regulation of the *c3566* operon by C3564/C3565 within murine RAW264.7 macrophages. GFP activity was monitored in wild-type CFT073 and its mutants, all of which carried a P_c3566_-EGFP fusion plasmid, during infection of RAW264.7 cells. Representative confocal fluorescence micrographs are shown.
(TIF)

S7 Fig Role of CpxR and its potential interconnection with C3564/C3565 in hemolysis and *c3566*-*c3568*-*hlyCABD* expression in UPEC strain CFT073. (A) Hemolysis on blood agar. (B) qPCR analysis of mRNA levels of *c3566*-*c3568*-*hlyCABD*. The dashed lines represent a fold change cutoff of 2, and a fold change ≥2 was considered significant. *c3567* was used to represent the *c3566-c3568* operon, and *hlyCA* to represent the *hlyCABD* operon.
(TIF)

S8 Fig Role of OxyR and its potential interconnection with OrhK/OrhR in hemolysis and gene expression in UPEC strain CFT073. (A) Hemolysis on blood agar. (B) qPCR analysis of mRNA expression. The dashed lines represent a fold change cutoff of 2, and a fold change ≥2 was considered significant. *c3567* was used to represent the *c3566-c3568* operon, and *hlyA* to represent the *hlyCABD* operon.
(TIF)

## References

[1] Russo TA, Johnson JR. Medical and economic impact of extraintestinal infections due to *Escherichia coli*: focus on an increasingly important endemic problem. Microbes Infect. 2003;5(5):449–56. doi: 10.1016/s1286-4579(03)00049-2 . 12738001

[2] Urinary Tract Infections Molecular Pathogenesis and Clinical Management. In: Mobley HLT, Warren JW, editors. Washington D.C.: American Society for Microbiology; 1996.

[3] Kaper JB, Nataro JP, Mobley HL. Pathogenic Escherichia coli. Nat Rev Microbiol. 2004;2(2):123–40. doi: 10.1038/nrmicro818 . 15040260

[4] Olson PD, Hunstad DA. Subversion of Host Innate Immunity by Uropathogenic Escherichia coli. Pathogens. 2016;5(1). doi: 10.3390/pathogens5010002 . 26742078 PMC4810123

[5] Rosen H, Klebanoff SJ, Wang Y, Brot N, Heinecke JW, Fu X. Methionine oxidation contributes to bacterial killing by the myeloperoxidase system of neutrophils. Proc Natl Acad Sci U S A. 2009;106(44):18686–91. doi: 10.1073/pnas.0909464106 . 19833874 PMC2774013

[6] Mulvey MA, Schilling JD, Martinez JJ, Hultgren SJ. Bad bugs and beleaguered bladders: Interplay between uropathogenic Escherichia coli and innate host defenses. Proc Natl Acad Sci U S A. 2000;97(16):8829–35. doi: 10.1073/pnas.97.16.8829 10922042 PMC34019

[7] Christman MF, Storz G, Ames BN. OxyR, a positive regulator of hydrogen peroxide-inducible genes in Escherichia coli and Salmonella typhimurium, is homologous to a family of bacterial regulatory proteins. Proc Natl Acad Sci U S A. 1989;86(10):3484–8. doi: 10.1073/pnas.86.10.3484 . 2471187 PMC287162

[8] Seo SW, Kim D, Szubin R, Palsson BO. Genome-wide Reconstruction of OxyR and SoxRS Transcriptional Regulatory Networks under Oxidative Stress in Escherichia coli K-12 MG1655. Cell Rep. 2015;12(8):1289–99. doi: 10.1016/j.celrep.2015.07.043 26279566

[9] Donovan GT, Norton JP, Bower JM, Mulvey MA. Adenylate Cyclase and the Cyclic AMP Receptor Protein Modulate Stress Resistance and Virulence Capacity of Uropathogenic Escherichia coli. Infection and immunity. 2013;81(1):249–58. doi: 10.1128/IAI.00796-12 23115037 PMC3536135

[10] Hryckowian AJ, Welch RA. RpoS Contributes to Phagocyte Oxidase-Mediated Stress Resistance during Urinary Tract Infection by Escherichia coli CFT073. Mbio. 2013;4(1). doi: 10.1128/mBio.00023-13 23404396 PMC3573659

[11] Cornelis G, Boland A, Boyd A, Gueijen C, Iriarte M, Neyt C, et al. The virulence plasmid of Yersinia, an antihost genome. Microbiol Mol Biol R. 1998;62(4):1315–52. doi: 10.1128/MMBR.62.4.1315-1352.1998 . 9841674 PMC98948

[12] Voth DE, Ballard JD. Clostridium difficile toxins: mechanism of action and role in disease. Clin Microbiol Rev. 2005;18(2):247–63. doi: 10.1128/CMR.18.2.247-263.2005 . 15831824 PMC1082799

[13] Johnson JR. Virulence factors in Escherichia coli urinary tract infection. Clin Microbiol Rev. 1991;4(1):80–128. doi: 10.1128/CMR.4.1.80 . 1672263 PMC358180

[14] Marrs CF, Zhang L, Tallman P, Manning SD, Somsel P, Raz P, et al. Variations in 10 putative uropathogen virulence genes among urinary, faecal and peri-urethral Escherichia coli. J Med Microbiol. 2002;51(2):138–42. doi: 10.1099/0022-1317-51-2-138 . 11863265

[15] Marrs CF, Zhang L, Foxman B. Escherichia coli mediated urinary tract infections: are there distinct uropathogenic E. coli (UPEC) pathotypes? FEMS MicrobiolLett. 2005;252(2):183–90. doi: 10.1016/j.femsle.2005.08.028 . 16165319

[16] A MVM, Phan MD, Peters KM, Nhu NTK, Welch RA, Ulett GC, et al. Regulation of hemolysin in uropathogenic Escherichia coli fine-tunes killing of human macrophages. Virulence. 2018;9(1):967–80. doi: 10.1080/21505594.2018.1465786 . 29683762 PMC5989160

[17] Russo TA, Davidson BA, Genagon SA, Warholic NM, MacDonald U, Pawlicki PD, et al. E-coli virulence factor hemolysin induces neutrophil apoptosis and necrosis/lysis in vitro and necrosislysis and lung injury in a rat pneumonia model. Am J Physiol-Lung C. 2005;289(2):L207–L16. doi: 10.1152/ajplung.00482.2004 15805136

[18] Wiles TJ, Mulvey MA. The RTX pore-forming toxin alpha-hemolysin of uropathogenic Escherichia coli: progress and perspectives. Future Microbiol. 2013;8(1):73–84. doi: 10.2217/fmb.12.131 23252494 PMC3570152

[19] Dhakal BK, Mulvey MA. The UPEC pore-forming toxin alpha-hemolysin triggers proteolysis of host proteins to disrupt cell adhesion, inflammatory, and survival pathways. Cell host & microbe. 2012;11(1):58–69. doi: 10.1016/j.chom.2011.12.003 . 22264513 PMC3266558

[20] Nagamatsu K, Hannan TJ, Guest RL, Kostakioti M, Hadjifrangiskou M, Binkley J, et al. Dysregulation of Escherichia coli alpha-hemolysin expression alters the course of acute and persistent urinary tract infection. Proc Natl Acad Sci U S A. 2015;112(8):E871–80. doi: 10.1073/pnas.1500374112 . 25675528 PMC4345586

[21] Barbieri NL, Nicholson B, Hussein A, Cai W, Wannemuehler YM, Dell’Anna G, et al. FNR regulates expression of important virulence factors contributing to pathogenicity of uropathogenic *Escherichia coli*. Infect Immun. 2014;82(12):5086–98. doi: 10.1128/IAI.02315-14 . 25245807 PMC4249304

[22] Leeds JA, Welch RA. RfaH enhances elongation of Escherichia coli hlyCABD mRNA. Journal of bacteriology. 1996;178(7):1850–7. doi: 10.1128/jb.178.7.1850-1857.1996 . 8606157 PMC177878

[23] Tomenius H, Pernestig AK, Jonas K, Georgellis D, Mollby R, Normark S, et al. The *Escherichia coli* BarA-UvrY two-component system is a virulence determinant in the urinary tract. BMC Microbiol. 2006;6:27. doi: 10.1186/1471-2180-6-27 . 16529647 PMC1421404

[24] Nhu NTK, Phan MD, Forde BM, Murthy AMV, Peters KM, Day CJ, et al. Complex Multilevel Control of Hemolysin Production by Uropathogenic Escherichia coli. Mbio. 2019;10(5). doi: 10.1128/mBio.02248-19 . 31575773 PMC6775461

[25] Stock AM, Robinson VL, Goudreau PN. Two-component signal transduction. Annual review of biochemistry. 2000;69:183–215. doi: 10.1146/annurev.biochem.69.1.183 . 10966457

[26] Hoch JA. Two-component and phosphorelay signal transduction. Current opinion in microbiology. 2000;3(2):165–70. doi: 10.1016/s1369-5274(00)00070-9 . 10745001

[27] Cai W, Wannemuehler Y, Dell’anna G, Nicholson B, Barbieri NL, Kariyawasam S, et al. A novel two-component signaling system facilitates uropathogenic Escherichia coli’s ability to exploit abundant host metabolites. PLoS pathogens. 2013;9(6):e1003428. doi: 10.1371/journal.ppat.1003428 . 23825943 PMC3694859

[28] Alteri CJ, Mobley HL. Metabolism and Fitness of Urinary Tract Pathogens. Microbiology spectrum. 2015;3(3). doi: 10.1128/microbiolspec.MBP-0016-2015 . 26185076 PMC4510461

[29] Sagulenko V, Thygesen SJ, Sester DP, Idris A, Cridland JA, Vajjhala PR, et al. AIM2 and NLRP3 inflammasomes activate both apoptotic and pyroptotic death pathways via ASC. Cell Death Differ. 2013;20(9):1149–60. doi: 10.1038/cdd.2013.37 23645208 PMC3741496

[30] Shi J, Zhao Y, Wang K, Shi X, Wang Y, Huang H, et al. Cleavage of GSDMD by inflammatory caspases determines pyroptotic cell death. Nature. 2015;526(7575):660–5. doi: 10.1038/nature15514 . 26375003

[31] Aachoui Y, Sagulenko V, Miao EA, Stacey KJ. Inflammasome-mediated pyroptotic and apoptotic cell death, and defense against infection. Curr Opin Microbiol. 2013;16(3):319–26. doi: 10.1016/j.mib.2013.04.004 23707339 PMC3742712

[32] Gennaris A, Ezraty B, Henry C, Agrebi R, Vergnes A, Oheix E, et al. Repairing oxidized proteins in the bacterial envelope using respiratory chain electrons. Nature. 2015;528(7582):409–12. doi: 10.1038/nature15764 . 26641313 PMC4700593

[33] Gray MJ, Wholey WY, Jakob U. Bacterial responses to reactive chlorine species. Annu Rev Microbiol. 2013;67:141–60. doi: 10.1146/annurev-micro-102912-142520 . 23768204 PMC3891400

[34] Boschi-Muller S, Gand A, Branlant G. The methionine sulfoxide reductases: Catalysis and substrate specificities. Archives of biochemistry and biophysics. 2008;474(2):266–73. doi: 10.1016/j.abb.2008.02.007 . 18302927

[35] Levine RL, Mosoni L, Berlett BS, Stadtman ER. Methionine residues as endogenous antioxidants in proteins. Proc Natl Acad Sci U S A. 1996;93(26):15036–40. doi: 10.1073/pnas.93.26.15036 8986759 PMC26351

[36] Melnyk RA, Youngblut MD, Clark IC, Carlson HK, Wetmore KM, Price MN, et al. Novel mechanism for scavenging of hypochlorite involving a periplasmic methionine-rich Peptide and methionine sulfoxide reductase. Mbio. 2015;6(3):e00233–15. doi: 10.1128/mBio.00233-15 . 25968643 PMC4436054

[37] Slauch JM. How does the oxidative burst of macrophages kill bacteria? Still an open question. Molecular microbiology. 2011;80(3):580–3. doi: 10.1111/j.1365-2958.2011.07612.x 21375590 PMC3109634

[38] Mavromatis C, Bokil NJ, Totsika M, Kakkanat A, Schaale K, Cannistraci CV, et al. The co-transcriptome of uropathogenic Escherichia coli-infected mouse macrophages reveals new insights into host-pathogen interactions. Cellular microbiology. 2015;17(5):730–46. doi: 10.1111/cmi.12397 25410299 PMC4950338

[39] Adachi Y, Kindzelskii AL, Petty AR, Huang JB, Maeda N, Yotsumoto S, et al. IFN-gamma primes RAW264 macrophages and human monocytes for enhanced oxidant production in response to CpG DNA via metabolic signaling: Roles of TLR9 and myeloperoxidase trafficking. J Immunol. 2006;176(8):5033–40. doi: 10.4049/jimmunol.176.8.5033 16585600

[40] Ezraty B, Bos J, Barras F, Aussel L. Methionine sulfoxide reduction and assimilation in Escherichia coli: New role for the biotin sulfoxide reductase BisC. Journal of bacteriology. 2005;187(1):231–7. doi: 10.1128/JB.187.1.231-237.2005 15601707 PMC538846

[41] Velasco E, Wang S, Sanet M, Fernandez-Vazquez J, Jove D, Glaria E, et al. A new role for Zinc limitation in bacterial pathogenicity: modulation of alpha-hemolysin from uropathogenic Escherichia coli. Scientific reports. 2018;8(1):6535. doi: 10.1038/s41598-018-24964-1 . 29695842 PMC5916954

[42] Nagy G, Altenhoefer A, Knapp O, Maier E, Dobrindt U, Blum-Oehler G, et al. Both alpha-haemolysin determinants contribute to full virulence of uropathogenic Escherichia coli strain 536. Microbes Infect. 2006;8(8):2006–12. doi: 10.1016/j.micinf.2006.02.029 . 16787757

[43] Salipante SJ, Roach DJ, Kitzman JO, Snyder MW, Stackhouse B, Butler-Wu SM, et al. Large-scale genomic sequencing of extraintestinal pathogenic Escherichia coli strains. Genome Res. 2015;25(1):119–28. doi: 10.1101/gr.180190.114 . 25373147 PMC4317167

[44] Ristow LC, Welch RA. Hemolysin of uropathogenic Escherichia coli: A cloak or a dagger? Biochimica et biophysica acta. 2016;1858(3):538–45. doi: 10.1016/j.bbamem.2015.08.015 . 26299820

[45] Cookson BT, Brennan MA. Pro-inflammatory programmed cell death. Trends Microbiol. 2001;9(3):113–4. doi: 10.1016/s0966-842x(00)01936-3 . 11303500

[46] Kayagaki N, Stowe IB, Lee BL, O’Rourke K, Anderson K, Warming S, et al. Caspase-11 cleaves gasdermin D for non-canonical inflammasome signalling. Nature. 2015;526(7575):666–71. doi: 10.1038/nature15541 . 26375259

[47] Ding J, Wang K, Liu W, She Y, Sun Q, Shi J, et al. Pore-forming activity and structural autoinhibition of the gasdermin family. Nature. 2016;535(7610):111–6. doi: 10.1038/nature18590 . 27281216

[48] Broz P, Pelegrin P, Shao F. The gasdermins, a protein family executing cell death and inflammation. Nature reviews Immunology. 2020;20(3):143–57. doi: 10.1038/s41577-019-0228-2 . 31690840

[49] Ashida H, Mimuro H, Ogawa M, Kobayashi T, Sanada T, Kim M, et al. Cell death and infection: a double-edged sword for host and pathogen survival. The Journal of cell biology. 2011;195(6):931–42. doi: 10.1083/jcb.201108081 . 22123830 PMC3241725

[50] Bien J, Sokolova O, Bozko P. Role of Uropathogenic Escherichia coli Virulence Factors in Development of Urinary Tract Infection and Kidney Damage. International journal of nephrology. 2012;2012:681473. doi: 10.1155/2012/681473 . 22506110 PMC3312279

[51] Smith YC, Rasmussen SB, Grande KK, Conran RM, O’Brien AD. aHemolysin of uropathogenic Escherichia coli evokes extensive shedding of the uroepithelium and hemorrhage in bladder tissue within the first 24 hours after intraurethral inoculation of mice. Infection and immunity. 2008;76(7):2978–90. doi: 10.1128/IAI.00075-08 18443089 PMC2446707

[52] Datsenko KA, Wanner BL. One-step inactivation of chromosomal genes in *Escherichia coli* K-12 using PCR products. Proc Natl Acad Sci U S A. 2000;97(12):6640–5. doi: 10.1073/pnas.120163297 . 10829079 PMC18686

[53] Johnson TJ, Wannemuehler Y, Doetkott C, Johnson SJ, Rosenberger SC, Nolan LK. Identification of minimal predictors of avian pathogenic *Escherichia coli* virulence for use as a rapid diagnostic tool. J Clin Microbiol. 2008;46(12):3987–96. doi: 10.1128/JCM.00816-08 . 18842938 PMC2593276

[54] Yamamoto K, Hirao K, Oshima T, Aiba H, Utsumi R, Ishihama A. Functional characterization in vitro of all two-component signal transduction systems from *Escherichia coli*. J Biol Chem. 2005;280(2):1448–56. doi: 10.1074/jbc.M410104200 . 15522865

[55] Crane JK, Majumdar S, Pickhardt DF. Host cell death due to enteropathogenic Escherichia coli has features of apoptosis. Infection and immunity. 1999;67(5):2575–84. doi: 10.1128/IAI.67.5.2575-2584.1999 10225923 PMC116006

[56] Zhang X, Li YX, Li BJ, Mao Y, Wu X, Zou XH, et al. Three supplementary methods for analyzing cytotoxicity of Escherichia coli O157:H7. Journal of microbiological methods. 2016;120:34–40. doi: 10.1016/j.mimet.2015.11.011 26593448

[57] Tsou AM, Zhu J. Quorum sensing negatively regulates hemolysin transcriptionally and posttranslationally in Vibrio cholerae. Infection and immunity. 2010;78(1):461–7. doi: 10.1128/IAI.00590-09 . 19858311 PMC2798175

[58] Sheehan LM, Budnick JA, Fyffe-Blair J, King KA, Settlage RE, Caswell CC. The Endoribonuclease RNase E Coordinates Expression of mRNAs and Small Regulatory RNAs and Is Critical for the Virulence of Brucella abortus. Journal of bacteriology. 2020;202(20). doi: 10.1128/JB.00240-20 32747427 PMC7515240

[59] Pfaffl MW. A new mathematical model for relative quantification in real-time RT-PCR. Nucleic Acids Res. 2001;29(9):e45. doi: 10.1093/nar/29.9.e45 . 11328886 PMC55695

[60] Xu N, Chu YL, Chen HL, Li XX, Wu Q, Jin L, et al. Rice transcription factor OsMADS25 modulates root growth and confers salinity tolerance via the ABA-mediated regulatory pathway and ROS scavenging. Plos Genet. 2018;14(10). doi: 10.1371/journal.pgen.1007662 30303953 PMC6197697

[61] Cai W, Cai X, Yang Y, Yan S, Zhang H. Transcriptional Control of Dual Transporters Involved in alpha-Ketoglutarate Utilization Reveals Their Distinct Roles in Uropathogenic Escherichia coli. Front Microbiol. 2017;8:275. doi: 10.3389/fmicb.2017.00275 . 28270808 PMC5318444

[62] Wan BS, Zhang QF, Ni JJ, Li SX, Wen DH, Li J, et al. Type VI secretion system contributes to Enterohemorrhagic Escherichia coli virulence by secreting catalase against host reactive oxygen species (ROS). PLoS pathogens. 2017;13(3). doi: 10.1371/journal.ppat.1006246 28288207 PMC5363993

[63] Sukumaran SK, Shimada H, Prasadarao NV. Entry and intracellular replication of Escherichia coli K1 in macrophages require expression of outer membrane protein A. Infection and immunity. 2003;71(10):5951–61. doi: 10.1128/IAI.71.10.5951-5961.2003 14500515 PMC201085

[64] Cieza RJ, Hu J, Ross BN, Sbrana E, Torres AG. The IbeA Invasin of Adherent-Invasive Escherichia coli Mediates Interaction with Intestinal Epithelia and Macrophages. Infection and immunity. 2015;83(5):1904–18. doi: 10.1128/IAI.03003-14 25712929 PMC4399045

[65] Srikumar S, Kroger C, Hebrard M, Colgan A, Owen SV, Sivasankaran SK, et al. RNA-seq Brings New Insights to the Intra-Macrophage Transcriptome of Salmonella Typhimurium. PLoS pathogens. 2015;11(11). doi: 10.1371/journal.ppat.1005262 26561851 PMC4643027

[66] Schaale K, Peters KM, Murthy AM, Fritzsche AK, Phan MD, Totsika M, et al. Strain-and host species-specific inflammasome activation, IL-1 beta release, and cell death in macrophages infected with uropathogenic Escherichia coli. Mucosal Immunol. 2016;9(1):124–36. doi: 10.1038/mi.2015.44 25993444

[67] Kaihami GH, Almeida JR, Santos SS, Netto LE, Almeida SR, Baldini RL. Involvement of a 1-Cys peroxiredoxin in bacterial virulence. PLoS pathogens. 2014;10(10):e1004442. doi: 10.1371/journal.ppat.1004442 . 25329795 PMC4199769

