# Supplementary material for: A previously uncharacterized two-component signaling system in uropathogenic Escherichia coli coordinates protection against host-derived oxidative stress with activation of hemolysin-mediated host cell pyroptosis
Source: PLoS Pathog. 2021 Oct 15;17(10):e1010005. doi: 10.1371/journal.ppat.1010005 (PMC8550376; doi:10.1371/journal.ppat.1010005)
Supplement: S1 Table — (DOC) [file ppat.1010005.s001.doc]

**S1 Table.** Strains and plasmids used in this study.

| **Bacterial strains and plasmids** | **Genotype/relevant characteristics** | **Source or Reference** |
| --- | --- | --- |
| **Bacterial strains** |  |  |
| *E. coli* DH5α | Plasmid propagation strain | Invitrogen |
| *E. coli* S17-λpir | RK2 *tra* regulon, *pir*, host for *pir-*dependent plasmids |  |
| UPEC CFT073 | Blood isolate from a patient with acute pyelonephritis |  |
| ∆*hlyA* | CFT073∆*lacZYA*∆*hlyA* | This study |
| ∆*c3564* | CFT073∆*lacZYA*∆*c3564* | This study |
| ∆*c3565* | CFT073∆*lacZYA*∆*c3565* | This study |
| ∆*c3564*/c35*65* | CFT073∆*lacZYA*∆*c3564*–*65* | This study |
| *∆c3564*-c35*68* | CFT073∆*lacZYA*∆*c3564*–*68* | This study |
| *∆c3564*–*hlyC-350* | CFT073∆*lacZYA∆c3564*–*hlyC-350* | This study |
| *∆c3564*–*hlyC-250* | CFT073∆*lacZYA∆c3564*–*hlyC-250* | This study |
| *∆c3564*–*hlyC-100* | CFT073∆*lacZYA∆c3564*–*hlyC-100* | This study |
| *∆c3566*-c35*68* | CFT073∆*lacZYA*∆*c3566*–*68* | This study |
| *∆c3566*–*hlyC-350* | CFT073∆*lacZYA∆c3566*–*hlyC-350* | This study |
| *∆c3566*–*hlyC-250* | CFT073∆*lacZYA∆c3566*–*hlyC-250* | This study |
| *∆c3566*–*hlyC-100* | CFT073∆*lacZYA∆c3566*–*hlyC-100* | This study |
| *∆c3568*–*hlyC-350* | CFT073∆*lacZYA∆c3568*–*hlyC-350* | This study |
| *∆c3568*–*hlyC-250* | CFT073∆*lacZYA∆c3568*–*hlyC-250* | This study |
| *∆c3568*–*hlyC-100* | CFT073∆*lacZYA∆c3568*–*hlyC-100* | This study |
| ∆*c3566* | CFT073∆*lacZYA*∆*c3566* | This study |
| ∆*c3567* | CFT073∆*lacZYA*∆*c3567* | This study |
| ∆*c3568* | CFT073∆*lacZYA*∆*c3568* | This study |
| ∆*c3566*/c35*67* | CFT073∆*lacZYA*∆*c3566*–*67* | This study |
| *∆c3567*/c35*68* | CFT073∆*lacZYA*∆*c3567*–*68* | This study |
| *∆P3566* | CFT073∆*lacZYA∆P3566* | This study |
| Δ*c3564*–*P3566*::*Pcm* | CFT073∆*lacZYA*Δ*c3564*–*P3566*::*Pcm* | This study |
| Δ*c3564*–*PhlyC*::*Pcm* | CFT073∆*lacZYA*Δ*c3564*–*PhlyC*::*Pcm* | This study |
| CFT*hlyA** | *hlyA* partial deletion mutant strain of CFT073 (Δ amino acids 564-936) | This study |
| **Plasmids** |  |  |
| pMAL-c2X | expression vector | New England Biolabs |
| pET21-a | expression vector | Novagen |
| pGEX-6P-3 | expression vector | GE Healthcare |
| pGEX-6P-3-c3564 | pGEX-6P-3 carrying partial *c3564* | This study |
| pGEX-6P-3-Mutc3564 | pGEX-6P-3 carrying partial C3564H278A | This study |
| pMAL-*c3564*/c35*65* | pMAL-c2x carrying *c3564*–*65* under the control of Ptac | This study |
| pMAL-*c3566*-c35*68* | pMAL-c2x carrying *c3566*–*68* under the control of Ptac | This study |
| pMAL-*c3564* | pMAL-c2x carrying *c3564* under the control of Ptac | This study |
| pMAL-*c3565* | pMAL-c2x carrying *c3565* under the control of Ptac | This study |
| pET21-*c3565* | pET21-a expression vector containing *c3565* gene | This study |
| pET21-*hlyA* | pET21-a expression vector containing *hlyA* gene | This study |
| pGEN-MCS | low copy plasmid for complementation |  |
| pHlyCABD | The entire *hlyCABD* cluster plus 1 kb upstream carryied in pGEN-MCS | This study |
| pGEN- P_CmR_ | pGEN-MCS carrying the promoter region of *cat* gene from pKD3 (P_CmR_) | This study |
| p*c3564*/c35*65* | pGEN-MCS carrying *c3564–65* coding region under the control of the native promoter | This study |
| p*c3564* | pGEN-P_CmR_ carrying *c3564* coding region under the control of P_CmR_ | This study |
| p*c3565* | pGEN-MCS carrying *c3565* coding region under the control of the native promoter | This study |
| p*c3566* | pGEN-MCS carrying *c3566* coding region under the control of the native promoter | This study |
| pc35*67* | pGEN-P_CmR_ carrying *c3567* coding region under the control of P_CmR_ | This study |
| p*c3566*–c35*67* | pGEN-MCS carrying *c3566–67* coding region under the control of the native promoter | This study |
| p*c3564*–*hlyC* | pGEN-MCS carrying *c3564*–*hlyC* coding region | This study |
| pEGFP-Prom/hlyC | pEGFP plasmid with promoter replaced with the promoter of *hlyC* | This study |
| pEGFP-Prom/ NC | pEGFP plasmid with promoter replaced with NC sequence | This study |
| pEGFP-Prom/c3566 | pEGFP plasmid with promoter replaced with the promoter of *c3566* | This study |
| pKD3 | template for λ-Red Chl^r^ cassette |  |
| pKD4 | template for λ-Red Kan^r^ cassette |  |
| pCP20 | encodes FLP recombinase for removal of resistance cassette |  |
| pKD46 | λ-Red recombinase expression |  |

1. Simon R, Priefer U, Puhler A. A Broad Host Range Mobilization System for In Vivo Genetic Engineering: Transposon Mutagenesis in Gram Negative Bacteria. Nat Biotech. 1983;1(9):784-91.

2. Welch RA, Burland V, Plunkett G, 3rd, Redford P, Roesch P, Rasko D, et al. Extensive mosaic structure revealed by the complete genome sequence of uropathogenic *Escherichia coli*. Proc Natl Acad Sci U S A. 2002;99(26):17020-4. PubMed PMID: 12471157.

3. Lane MC, Alteri CJ, Smith SN, Mobley HL. Expression of flagella is coincident with uropathogenic Escherichia coli ascension to the upper urinary tract. Proc Natl Acad Sci U S A. 2007;104(42):16669-74. doi: 10.1073/pnas.0607898104. PubMed PMID: 17925449.

4. Datsenko KA, Wanner BL. One-step inactivation of chromosomal genes in Escherichia coli K-12 using PCR products. Proc Natl Acad Sci U S A. 2000;97(12):6640-5. Epub 2000/06/01. doi: 10.1073/pnas.120163297. PubMed PMID: 10829079.
