# Supplementary material for: A previously uncharacterized two-component signaling system in uropathogenic Escherichia coli coordinates protection against host-derived oxidative stress with activation of hemolysin-mediated host cell pyroptosis
Source: PLoS Pathog. 2021 Oct 15;17(10):e1010005. doi: 10.1371/journal.ppat.1010005 (PMC8550376; doi:10.1371/journal.ppat.1010005)
Supplement: S2 Table — (DOCX) [file ppat.1010005.s002.docx]

**S2 Table.** Oligonucleotides used in this study.

| **Primers** | **Sequence (5'-3')** |
| --- | --- |
| **For Cloning** |  |
| pGEX-6P-c3564-P1 | TCCGAATTCCTACTGGGTAACCCGTCCCAT |
| pGEX-6P-c3564-P2 | CAGGTCGACTCATTATAATGGAAGAAAAAAACG |
| pGEX-6P-c3564-mut-P1 | GGAGATATTGGCAATAAATTCAC |
| pGEX-6P-c3564-mut-P2 | TTTATTGCCAATATCTCCGCTGATTTACGGACGCCATTAAC |
| pGEX-6P-c3564-mut-P3 | CTGTAGGTATCTCAGTTCGGTGTAGGTCGTTCGCTC |
| pGEX-6P-c3564-mut-P4 | AACTGAGATACCTACAGCGTG |
| pET-hlyA-F | GGAATTCCATATGCAGAAGCAAGTCTTTGACCCAT |
| pET-hlyA-R | GAACCGCTCGAGCAGCGTATCATTACCTTTATCACC |
| pET-c3565-F | TCGCGGATCCGAATTCATGAATAACGTAAAAAAAATACTG |
| pET-c3565-R | GACGGAGCTCTTATCGCATTCCTGCATTGT |
| phlyCABD-F | TGAAGCTTGGTACCGGGATCCGTTGAGAACTTAAAATTACGTTACGATAAA |
| phlyCABD-R | GAAAGGGCAGATTGTGTCGACTTAACGCTCATGTAAACTTTCTGTTACA |
| pMAL-c3566–68-F | ACGCCATATGCAGCCATTACCGTTAAAACA |
| pMAL-c3566–68-R | ACGCGGATCCTGCGCAGAAATGATGCTTACG |
| pGEN-P_CmR_-F | ACGCGAATTCTAGGAACTTCGGCGCGCCTA |
| pGEN-P_CmR_-R | ACGCCATATGTTTAGCTTCCTTAGCTCCTGA |
| c3564-hlyC-F | ACGCGTCGACTTTATCGACCTCACACGAACA |
| c3564-hlyC-R | ACGCGTCGACTATCCCGGGTTAATAAAGCAT |
| pMAL-c3564–65-F | GGATAACATATGATGAATAACGTAAAAAAAATACTG |
| pMAL-c3564–65-R | CGCCTGATGTTAGGATCCAAGAAAAAAACGAAAGCA |
| pEGFP-Prom/hlyC-P1 | CTGCATTAATGAATCGGC |
| pEGFP-Prom/hlyC-P2 | CCGATTCATTAATGCAGATATTTTAGAGTATACTTGCGCACCCG |
| pEGFP-Prom/hlyC-P3 | GCCCTTGCTCACCATTGCGGTGGCAGGTAAAAAAAAG |
| pEGFP-Prom/hlyC-P4 | ATGGTGAGCAAGGGCGAG |
| pEGFP-Prom/NC-P1 | CTGCATTAATGAATCGGC |
| pEGFP-Prom/NC -P2 | CCGATTCATTAATGCAGGACTGATCTTTCAACAGAATACTC |
| pEGFP-Prom/NC -P3 | GCCCTTGCTCACCATCGATTCAAAAAACTTGGATAC |
| pEGFP-Prom/NC -P4 | ATGGTGAGCAAGGGCGAG |
| pEGFP-Prom/c3566-P1 | CTGCATTAATGAATCGGC |

| pEGFP-Prom/c3566-P2 | CCGATTCATTAATGCAGGCAGATAACGGGTCATCTG |
| --- | --- |
| pEGFP-Prom/c3566-P3 | GCCCTTGCTCACCATTTGTTTTAACGGTAATGGC |
| pEGFP-Prom/c3566-P4 | ATGGTGAGCAAGGGCGAG |
|  |  |
| **For qPCR** |  |
| c3564-rP1 | CGCTTTACCTGAGGGATGA |
| c3564-rP2 | ATACCCGACAGCAGACCAC |
| c3565-rP1 | CATATCAACCGTCTTCGTG |
| c3565-rP2 | CATTCCTGCATTGTCAACT |
| c3566-rP1 | TGGCTTGGTGGTGCGTTAC |
| c3566-rP2 | GGTCCATGACAGCGGGAAA |
| c3567-rP1 | ATGATGCTGACTGGCTGTG |
| c3567-rP2 | AAGGGAATGGAGTGGTAATGT |
| c3568-rP1 | TCATGTTCAATGCTCAGGC |
| c3568-rP2 | TTTTCATCCCATCCGTTTT |
| Hly-c-rp1 | ATTGACTGGATTGCTCCTT |
| Hly-c-rp2 | CCTCCGTGAAATTCTGATA |
| Hly-a-rp1 | GTGACTATCTTTGCACCACAA |
| Hly-a-rp2 | CACTGCCTGCCTTTCCTAA |
| Hly-b-rp1 | AAGTCGGATTGATGTTGAG |
| Hly-b-rp2 | AAATTACGGATCTGGTCTA |
| Hly-d-rp1 | GCCTTTCCTTACACCCGATA |
| Hly-d-rp2 | TGCAGTGACAGCCATACCC |
|  |  |
| **For Deletion** |  |
| Del-c3564-F | CTTTTACAAAGGCAGATACACATAACCACCCCAAAATATGCCGCCTGATGgtgtaggctggagctgcttcga |
| Del-c3564-R | TCTGGGGAAAAGGGTATAGGTTTTCAGTTGACAATGCAGGAATGCGATAAcatatgaatatcctccttag |
| Del-c3565-F | ACTGTAAATACTAGGCTTAACCTCTGGCTTAATGTCAGGCTACAATTCATgtgtaggctggagctgcttcga |
| Del-c3565-R | CATCGTCAAATGCTGGGGTAAAATTCAGATAAAGAATATGTGGATAACTTcatatgaatatcctccttag |
| Del-c3564/65-F | CTTTTACAAAGGCAGATACACATAACCACCCCAAAATATGCCGCCTGATGgtgtaggctggagctgcttcga |
| Del-c3564/65-R | CATCGTCAAATGCTGGGGTAAAATTCAGATAAAGAATATGTGGATAACTTcatatgaatatcctccttag |
| Del-hlyA-F | AAAAACAAGACAGATTTCAATTTTTCATTAACAGGTTAAGAGATAATTAAgtgtaggctggagctgcttcga |
| Del-hlyA-R | AATCTTATGTGGCACAGCCCAGTAAGATTGCTATTATTTAAATTAATAAAcatatgaatatcctccttag |
| Del-c3564–68-F | CTTTTACAAAGGCAGATACACATAACCACCCCAAAATATGCCGCCTGATGgtgtaggctggagctgcttcga |
| Del-c3564–68-R | CGACAGAATATGATGTTTTATCGTAACGTAATTTTAAGTTCTCAACTTATcatatgaatatcctccttag |
| Del-c3564–hlyC-350-F | CTTTTACAAAGGCAGATACACATAACCACCCCAAAATATGCCGCCTGATGgtgtaggctggagctgcttcga |
| Del-c3564–hlyC-350-R | CCAATCAGCTGCCGAATGATGACCTGTGAGTTGTCATGTGAACCTCTCTTcatatgaatatcctccttag |
| Del-c3564–hlyC-250-F | CTTTTACAAAGGCAGATACACATAACCACCCCAAAATATGCCGCCTGATGgtgtaggctggagctgcttcga |
| Del-c3564–hlyC-250-R | AAACCATGCTATTGTATTCTCTTCAATATGCACATTCTAAAGAACTGTTAcatatgaatatcctccttag |
| Del-c3564–hlyC-100-F | CTTTTACAAAGGCAGATACACATAACCACCCCAAAATATGCCGCCTGATGgtgtaggctggagctgcttcga |
| Del-c3564–hlyC-100-R | TGAGAGAACGATATTAATCCTTTAAATATGTTTCTTGCATTTAGTTTCATcatatgaatatcctccttag |
| Del-c3566–hlyC-350-F | TGATGAACATTCCTTTCCATAATATACTTAGTTTACAGACAGAGATCACAgtgtaggctggagctgcttcga |
| Del-c3566–hlyC-350-R | CCAATCAGCTGCCGAATGATGACCTGTGAGTTGTCATGTGAACCTCTCTTcatatgaatatcctccttag |
| Del-c3566–hlyC-250-F | TGATGAACATTCCTTTCCATAATATACTTAGTTTACAGACAGAGATCACAgtgtaggctggagctgcttcga |
| Del-c3566–hlyC-250-R | AAACCATGCTATTGTATTCTCTTCAATATGCACATTCTAAAGAACTGTTAcatatgaatatcctccttag |
| Del-c3566–hlyC-100-F | TGATGAACATTCCTTTCCATAATATACTTAGTTTACAGACAGAGATCACAgtgtaggctggagctgcttcga |
| Del-c3566–hlyC-100-R | TGAGAGAACGATATTAATCCTTTAAATATGTTTCTTGCATTTAGTTTCATcatatgaatatcctccttag |
| Del-c3568–hlyC-350-F | ATGGGATGAAAAATGACCACATGGGTAAAAAAGACAATATGTCTCAATAAgtgtaggctggagctgcttcga |
| Del-c3568–hlyC-350-R | CCAATCAGCTGCCGAATGATGACCTGTGAGTTGTCATGTGAACCTCTCTTcatatgaatatcctccttag |
| Del-c3568–hlyC-250-F | ATGGGATGAAAAATGACCACATGGGTAAAAAAGACAATATGTCTCAATAAgtgtaggctggagctgcttcga |
| Del-c3568–hlyC-250-R | AAACCATGCTATTGTATTCTCTTCAATATGCACATTCTAAAGAACTGTTAcatatgaatatcctccttag |
| Del-c3568–hlyC-100-F | ATGGGATGAAAAATGACCACATGGGTAAAAAAGACAATATGTCTCAATAAgtgtaggctggagctgcttcga |
| Del-c3568–hlyC-100-R | TGAGAGAACGATATTAATCCTTTAAATATGTTTCTTGCATTTAGTTTCATcatatgaatatcctccttag |
| Del-c3566-F | ATGAACATTCCTTTCCATAATATACTTAGTTTACAGACAGAGATCACATAgtgtaggctggagctgcttcga |
| Del-c3566-R | CTGAACGGGTTTCCATAAAACCAGACCAGACAATAGCAGAGCAGCGCCATcatatgaatatcctccttag |
| Del-c3567-F | CTGACTATACTATTCGGTGGTTATGATACAGTGCGTTATATCCACTTTCTgtgtaggctggagctgcttcga |
| Del-c3567-R | CCTCCTCATTTTTAACAATTGTATCAACAACCACCAAACCAGTTATAACCcatatgaatatcctccttag |
| Del-c3568-F | ATAACTGGTTTGGTGGTTGTTGATACAATTGTTAAAAATGAGGAGGAACCgtgtaggctggagctgcttcga |
| Del-c3568-R | AAAACGACAGAATATGATGTTTTATCGTAACGTAATTTTAAGTTCTCAACcatatgaatatcctccttag |
| Del-c3566/67-F | ATGAACATTCCTTTCCATAATATACTTAGTTTACAGACAGAGATCACATAgtgtaggctggagctgcttcga |
| Del-c3566/67-R | CCTCCTCATTTTTAACAATTGTATCAACAACCACCAAACCAGTTATAACCcatatgaatatcctccttag |
| Del-c3567/68-F | CTGACTATACTATTCGGTGGTTATGATACAGTGCGTTATATCCACTTTCTgtgtaggctggagctgcttcga |
| Del-c3567/68-R | AAAACGACAGAATATGATGTTTTATCGTAACGTAATTTTAAGTTCTCAACcatatgaatatcctccttag |
| Del-c3566–68-F | ATGAACATTCCTTTCCATAATATACTTAGTTTACAGACAGAGATCACATAgtgtaggctggagctgcttcga |
| Del-c3566–68-R | AAAACGACAGAATATGATGTTTTATCGTAACGTAATTTTAAGTTCTCAACcatatgaatatcctccttag |
| Del-P3566-F | GCAGATTAAGCCGCAACAGAGCTGCAATATCATAATCATCTTCCATCAGCgtgtaggctggagctgcttcga |
| Del-P3566-R | ACAGAGGATGTATTTGTTTTAACGGTAATGGCTGCATTATGTGATCTCTGcatatgaatatcctccttag |
| Del-c3564–P3566::Pcm-F1 | CTTTTACAAAGGCAGATACACATAACCACCCCAAAATATGCCGCCTGATGgtgtaggctggagctgcttcga |
| Del-c3564–P3566::Pcm-R1 | tccgtcacaggtaggcgcgccatatgaatatcctccttag |
| Del-c3564–P3566::Pcm-F2 | ctaaggaggatattcatatggcgcgcctacctgtgacgga |
| Del-c3564–P3566::Pcm-R2 | CAACAGCGTAACCACAGAGGATGTATTTGTTTTAACGGTAATGGCTGCAttttagcttccttagctcctga |
| Del-c3564–PhlyC::Pcm-F1 | CTTTTACAAAGGCAGATACACATAACCACCCCAAAATATGCCGCCTGATGgtgtaggctggagctgcttcga |
| Del-c3564–PhlyC::Pcm-R1 | TCCGTCACAGGTAGGCGCGCCATATGAATATCCTCCTTAG |
| Del-c3564–PhlyC::Pcm-F2 | CTAAGGAGGATATTCATATGGCGCGCCTACCTGTGACGGA |
| Del-c3564–PhlyC::Pcm-R2 | CAGAGCCAGGATACATGCCCAAGAACCTCTAATGGATTGTTCATATTCAttttagcttccttagctcctga |
| Del-hlyA*-F | TGTTACGCCATTGTTAACTCCCGGTGAGGAAATTCGTGAAAGGAGGCAGTCCGGAgtgtaggctggagctgcttcga |
| Del-hlyA*-R | TATTATTACTCTGTTGATACTCAAGTGCCTTTTTAAGGGAATCTGGTGTGATTACcatatgaatatcctccttag |
| \| Del-oxyR-F \| \| --- \| \| Del-oxyR-R \| | \| GTTCGTTGCTATGCTACCTATCGCCGCGAACTATCGTGGCAATGGAGGATGGATAGTGTAGGCTGGAGCTGCTTCGA \| \| --- \| \| GGCGGAAGCCTATCGGGTAGCTGCGCTAAATGGCTTAAACCGCCTGTTTTAACACCATATGAATATCCTCCTTAG \| |
| \| cpxR-del-F \| \| --- \| \| cpxR-del-R \| | \| CTGCCTCGGAGGTATTTAAACAATGAATAAAATCCTGTTAGTTGATGATGACCGAgtgtaggctggagctgcttcga \| \| --- \| \| TCATGAAGCAGAAACCATCAGATAGCCGCGACCACGCAGGGTTTTAAACCACGGGcatatgaatatcctccttag \| |
| **For Mutation verification** |  |
| Check-c3564–65-F | CGACCTCACACGAACAACGA |
| Check-c3564–65-R | GCGAAGTGCCACAGTAACGC |
| Check-c3564-F | ACAGGTTATCCCGGTGTC |
| Check-c3564-R | TCAAAGCGTTGTTTCGTC |
| Check-c3565-F | TTTCAGATCATCCCTCAG |
| Check-c3565-R | GCTACAAATTCCCAGAGT |
| Check-c3566-F | TTTACCCCAGCATTTGACG |
| Check-c3566-R | CAGAAGGGAAAACTGAACG |
| Check-c3567-F | GCTCTGCTATTGTCTGGTCT |
| Check-c3567-R | TTTCATGGTTCCTCCTCAT |
| Check-c3568-F | TGGCGACAGCACTACATTC |
| Check-c3568-R | CCGATACAGAGCCTGACATT |
| Check-c3564–68-F | CGACCTCACACGAACAACGA |
| Check-c3564–68-R | TGCGCAGAAATGATGCTTACG |
| Check-c3564–hlyC-F | CGACCTCACACGAACAACGA |
| Check-c3564–hlyC-R | CCCGAAAGGAGCAATCCAGT |
| Check-P3566-F | TTGCCCCATGGCTTCCTGAC |
| Check-P3566-R | GCGAAGTGCCACAGTAACGC |
| Check-c3566–68-F | ATGGAAAAGGGTTTCGGTAGAT |
| Check-c3566–68-R | TGCGCAGAAATGATGCTTACG |
| Check-c3566–hlyC-F | TTGCCCCATGGCTTCCTGAC |
| Check-c3566–hlyC-R | CCCGAAAGGAGCAATCCAGT |
| Check-c3568–hlyC-F | TGAGCAAAACGGATGGGATGA |
| Check-c3568–hlyC-R | CCCGAAAGGAGCAATCCAGT |
| Check-hlyA-F | CTGGGCCAGTTCCCCATTAC |
| Check-hlyA-R | GGGGATTTCGTTGCTCCAGA |
| Check-hlyA*-F | GTGTCACCAGAAATGGAGAC |
| Check-hlyA*-R | ATTAAGATTATCCTGACTTCC |
|  |  |
| **For EMSA** |  |
| Prom-c3565-F | CAGCGTAACCACAGAGGATG |
| Prom-c3565-R | CGCAACAGAGCTGCAATATC |
| Control-c3565-F | ATGACGTGCAAACCAGAGCA |
| Control-c3565-R | GTCCTGGGACTGGAAATGGG |
| Prom-c3566-F | TCCCAGGTCTGCTTGTCTAGT |
| Prom-c3566-R | CCTGCGACAGTAACAGCAT |
| Control-c3566–67-F | TCTGCTGATTAATGCCACGA |
| Control-c3566–67-R | CAATGCACGCGGAACCAATA |
|  |  |
| **For 5'-RACE** |  |
| Race-RNA-adapter | UCAUACACAUACGAUUUAGGUGACACUAUAGAGCGGCCGCCUGCAGGAAA |
| Race-adapter-F | GCGCGAATTCACACATACGATTTAGGTGACACT |
| Race-GSP-c3566 | GTCAGTCGACGTGCCTGCGTAAGGGATTCT |
| MultiP PCR-c3568–hlyC-F | ACGGATGGGATGAAAAATGA |
| MultiP PCR-c3568–hlyC-R | TGCCCAAGAACCTCTAATGG |
|  |  |
| **For RT-PCR** |  |
| RT-c3566–c3567-F | CCCGCTGTCATGGACCTCAC |
| RT-c3566–c3567-R | TCACGTACCAGCCCCCGTAT |
| RT-c3567–c3568-F | CGCCGGACAGACGTCGTTTA |
| RT-c3567–c3568-R | TTGTGTCTGCAGCCTGAGCAT |
| RT-c3568–hlyC-F | TGAGCAAAACGGATGGGATGA |
| RT-c3568–hlyC-R | CCCGAAAGGAGCAATCCAGT |
| RT-hlyC–hlyA-F | CTGGGCCAGTTCCCCATTAC |
| RT-hlyC–hlyA-R | CTCTGCTGTGCCGAATACCT |
| RT-hlyA–hlyB-F | GTTGTACGGCAGTGAGGGAG |
| RT-hlyA–-hlyB-R | AGATTTCGCAGCAAGCAACC |
| RT-hlyB–hlyD-F | CTGGTTACGTCGTCAGGTGG |
| RT-hlyB–hlyD-R | AACGTATCAGCTTCAGCTCCC |
