# Supplementary figures and images for: A previously uncharacterized two-component signaling system in uropathogenic Escherichia coli coordinates protection against host-derived oxidative stress with activation of hemolysin-mediated host cell pyroptosis
Source: PLoS Pathog. 2021 Oct 15;17(10):e1010005. doi: 10.1371/journal.ppat.1010005 (PMC8550376; doi:10.1371/journal.ppat.1010005)

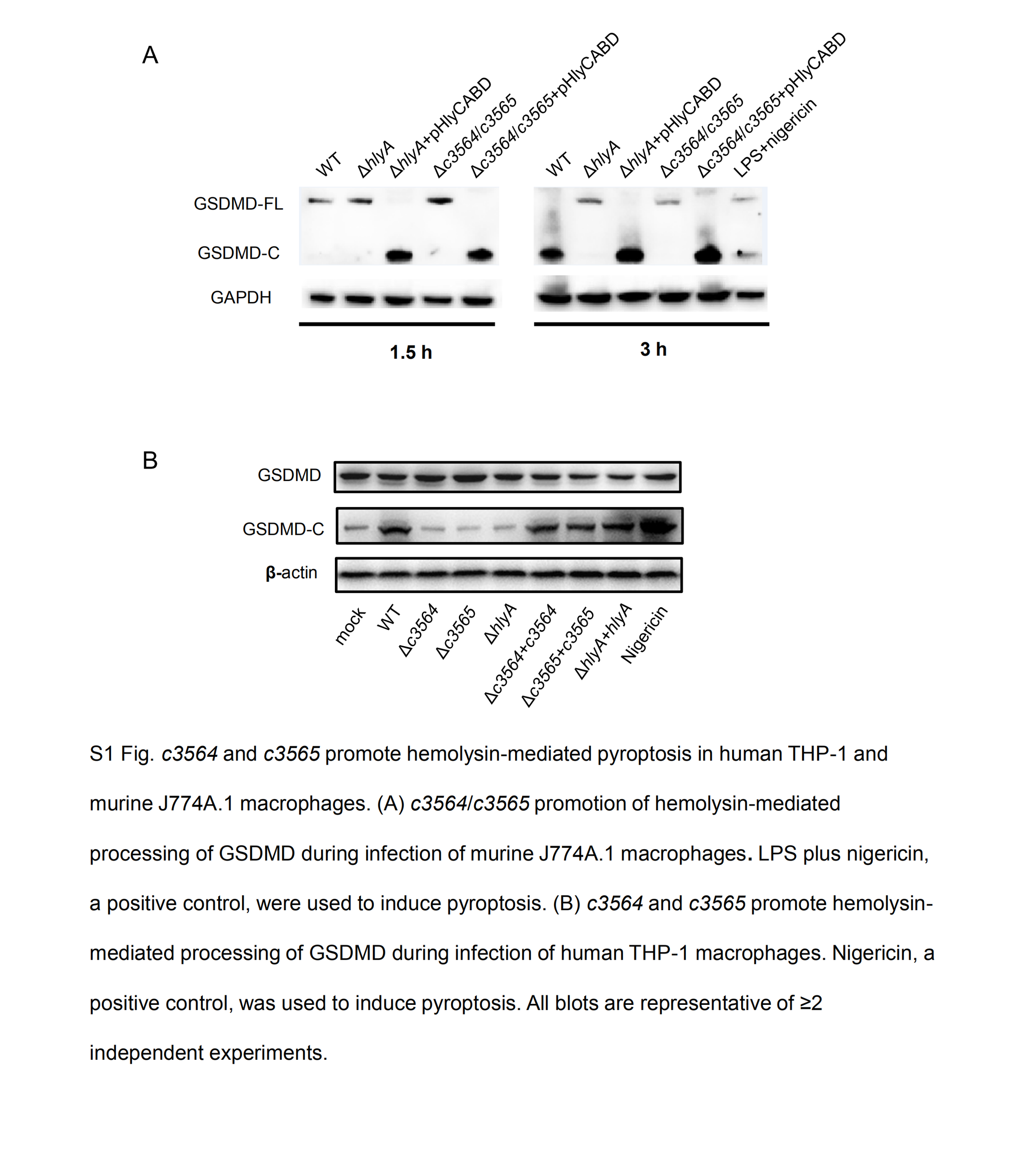

Supplement: S1 Fig — (A) c3564/c3565 promotion of hemolysin-mediated processing of GSDMD during infection of murine J774A.1 macrophages. LPS plus nigericin, a positive control, were used to induce pyroptosis. (B) c3564 and c3565 promote hemolysin-mediated processing of GSDMD during infection of human THP-1 macrophages. Nigericin, a positive control, was used to induce pyroptosis. All blots are representative of ≥2 independent experiments. (TIF) [file ppat.1010005.s004.tif]

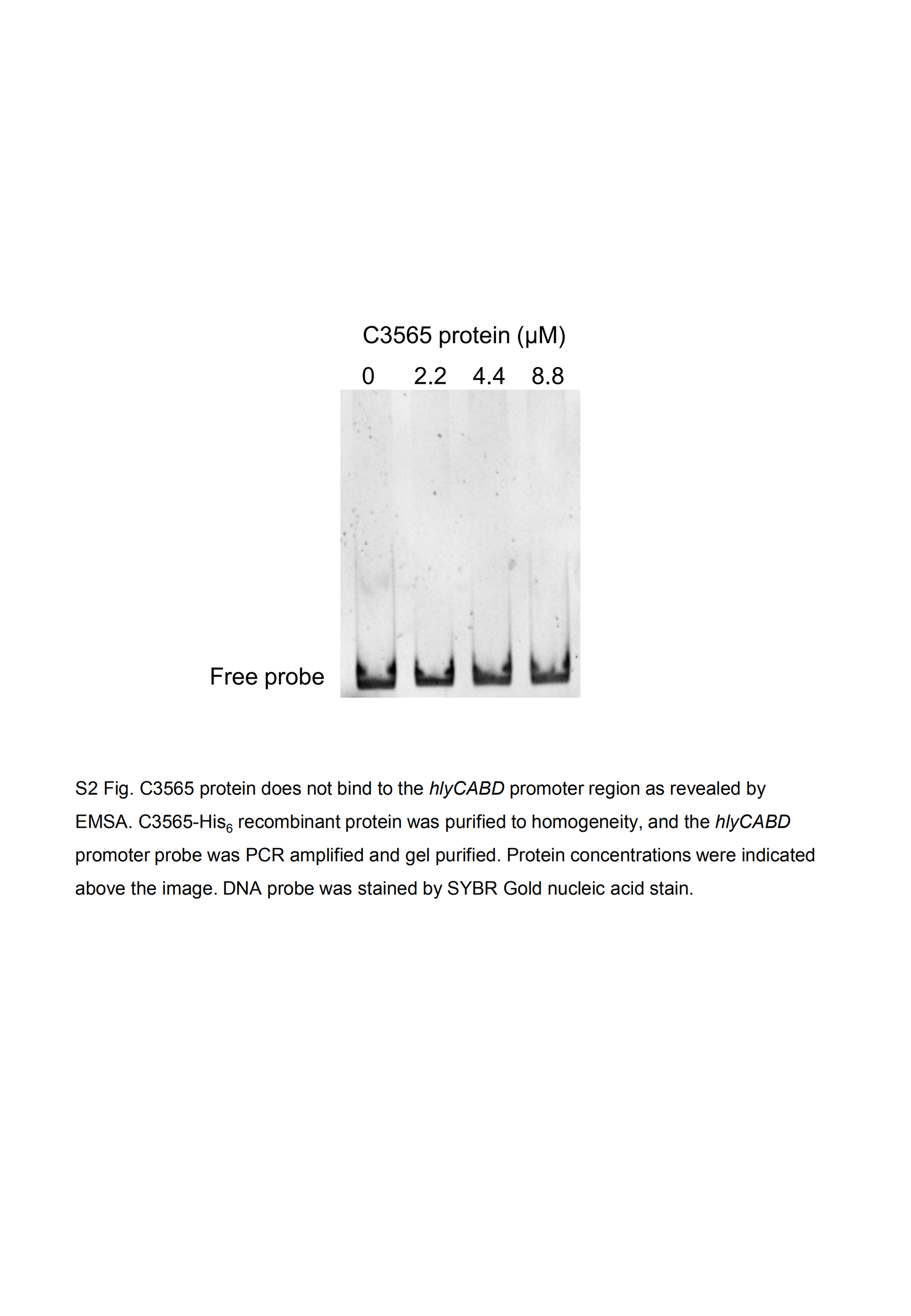

Supplement: S2 Fig — C3565-His6 recombinant protein was purified to homogeneity, and the hlyCABD promoter probe was PCR amplified and gel purified. Protein concentrations were indicated above the image. DNA probe was stained by SYBR Gold nucleic acid stain. (TIF) [file ppat.1010005.s005.tif]

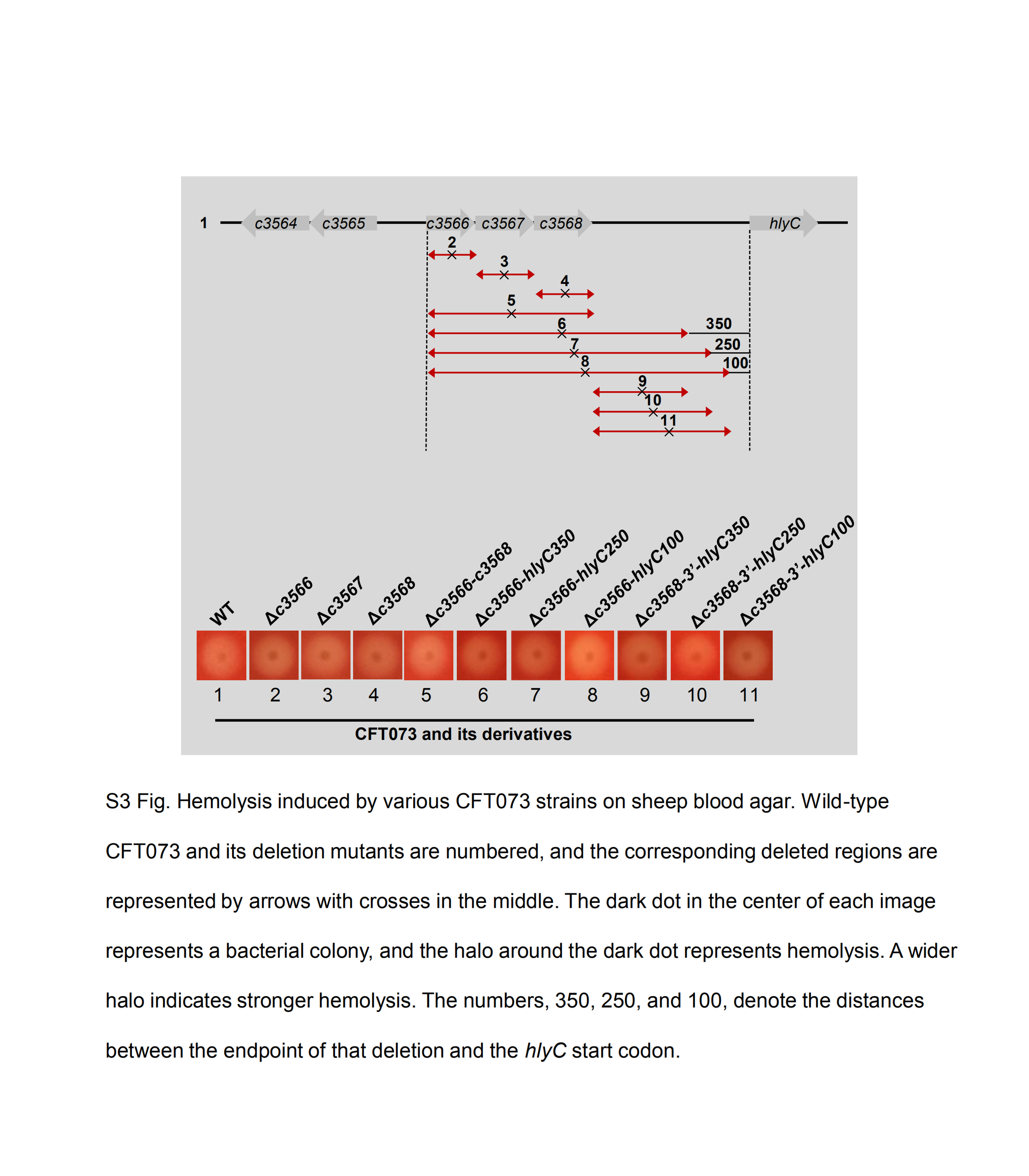

Supplement: S3 Fig — Hemolysis induced by various CFT073 strains on sheep blood agar. Wild-type CFT073 and its deletion mutants are numbered, and the corresponding deleted regions are represented by arrows with crosses in the middle. The dark dot in the center of each image represents a bacterial colony, and the halo around the dark dot represents hemolysis. A wider halo indicates stronger hemolysis. The numbers, 350, 250, and 100, denote the distances between the endpoint of that deletion and the hlyC start codon. (TIF) [file ppat.1010005.s006.tif]

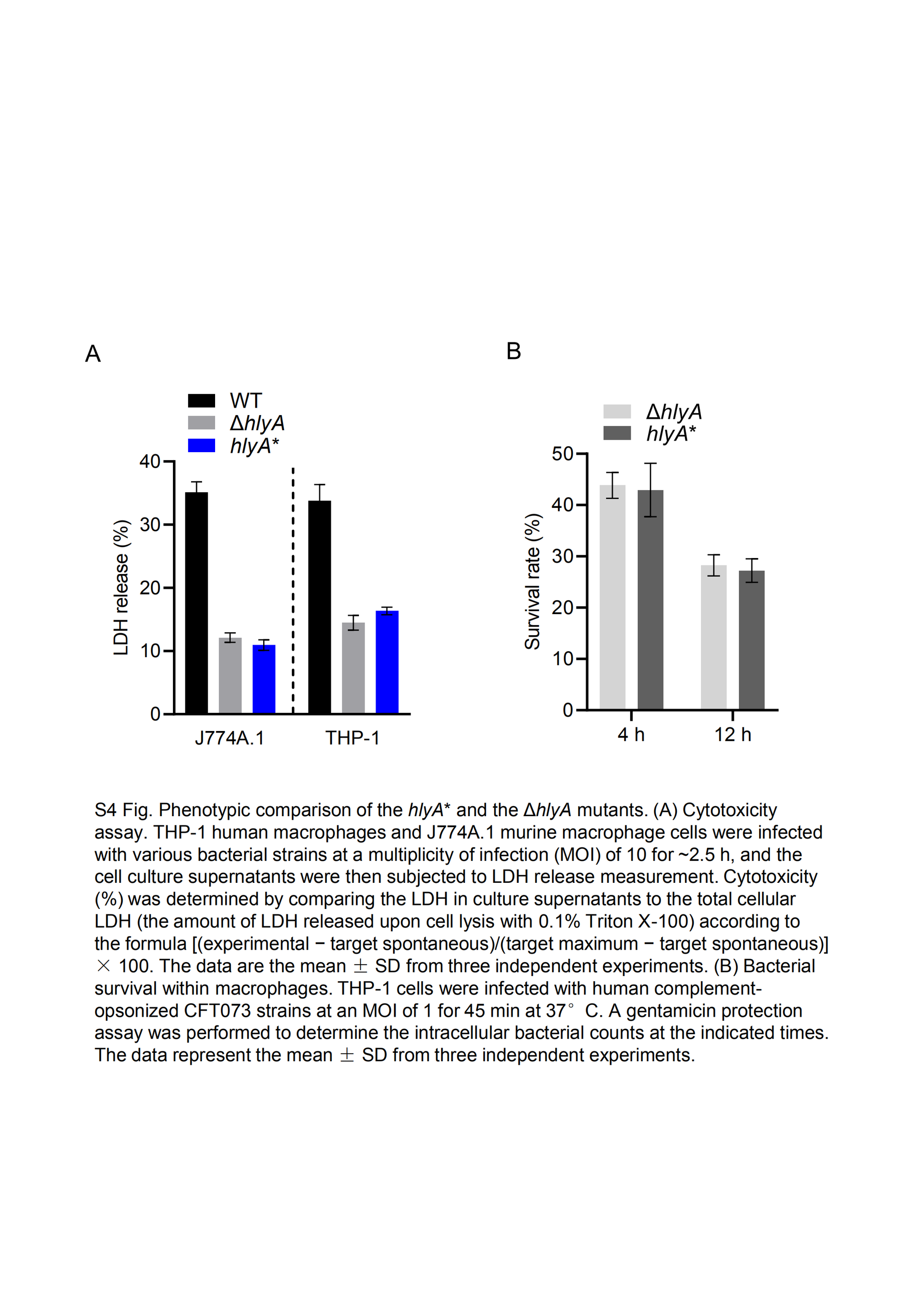

Supplement: S4 Fig — (A) Cytotoxicity assay. THP-1 human macrophages and J774A.1 murine macrophage cells were infected with various bacterial strains at a multiplicity of infection (MOI) of 10 for ~2.5 h, and the cell culture supernatants were then subjected to LDH release measurement. Cytotoxicity (%) was determined by comparing the LDH in culture supernatants to the total cellular LDH (the amount of LDH released upon cell lysis with 0.1% Triton X-100) according to the formula [(experimental − target spontaneous)/(target maximum − target spontaneous)] × 100. The data are the mean ± SD from three independent experiments. (B) Bacterial survival within macrophages. THP-1 cells were infected with human complement-opsonized CFT073 strains at an MOI of 1 for 45 min at 37°C. A gentamicin protection assay was performed to determine the intracellular bacterial counts at the indicated times. The data represent the mean ± SD from three independent experiments. (TIF) [file ppat.1010005.s007.tif]

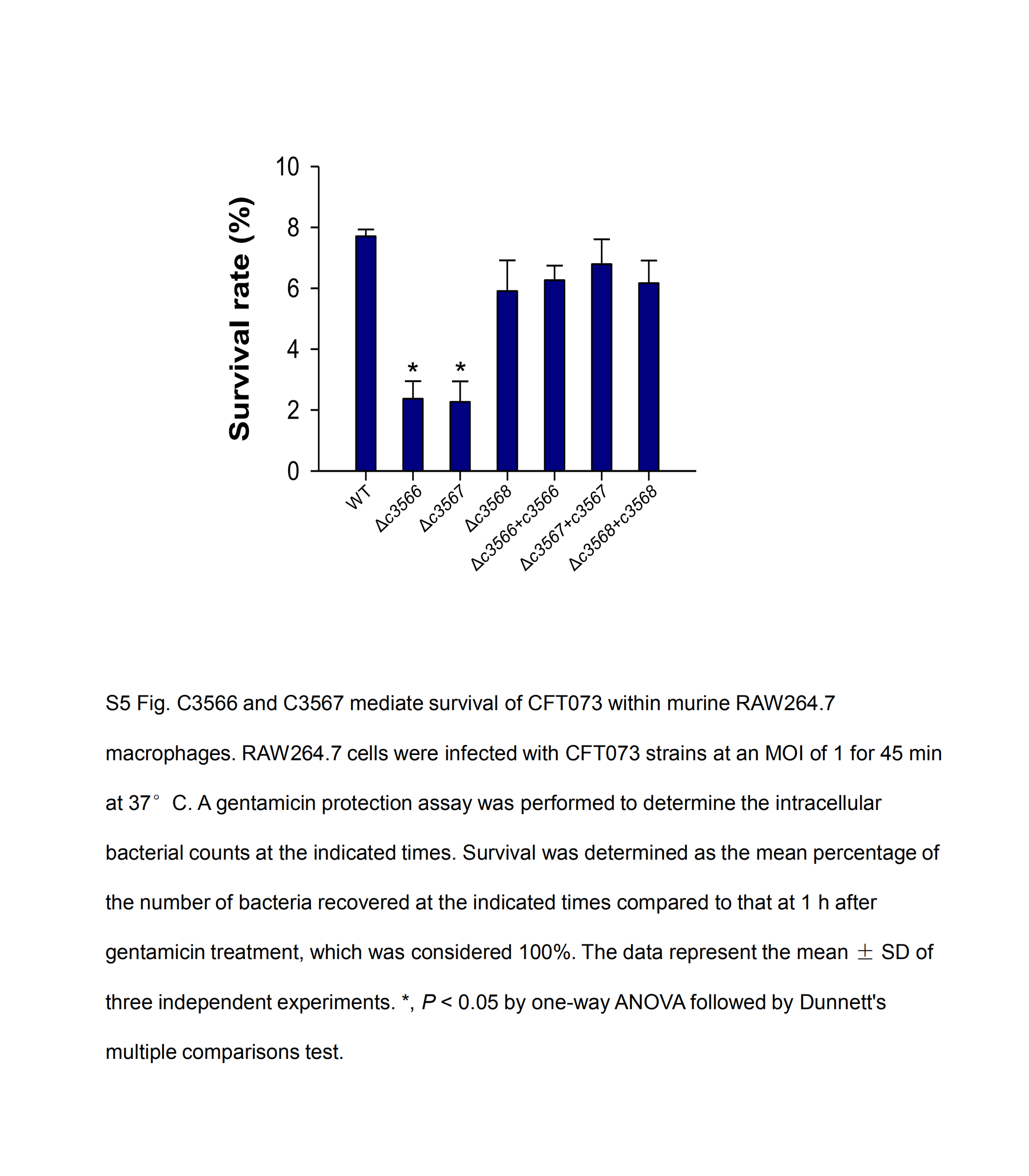

Supplement: S5 Fig — RAW264.7 cells were infected with CFT073 strains at an MOI of 1 for 45 min at 37°C. A gentamicin protection assay was performed to determine the intracellular bacterial counts at the indicated times. Survival was determined as the mean percentage of the number of bacteria recovered at the indicated times compared to that at 1 h after gentamicin treatment, which was considered 100%. The data represent the mean ±SD of three independent experiments. *, P < 0.05 by one-way ANOVA followed by Dunnett’s multiple comparisons test. (TIF) [file ppat.1010005.s008.tif]

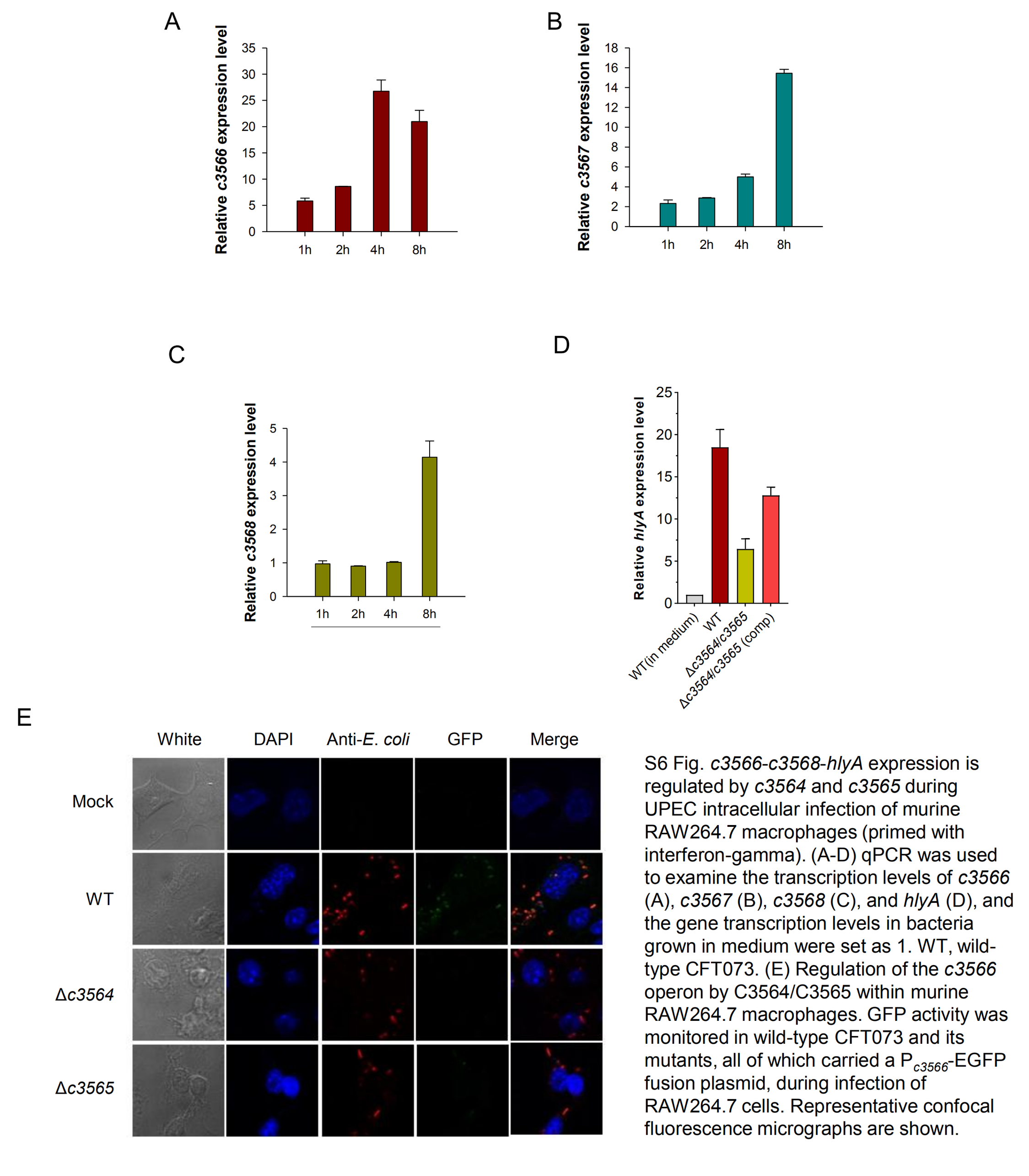

Supplement: S6 Fig — (A, B, C, and D) qPCR was used to examine the transcription levels of c3566 (A), c3567 (B), c3568 (C), and hlyA (D), and the gene transcription levels in bacteria grown in medium were set as 1. WT, wild-type CFT073. (E) Regulation of the c3566 operon by C3564/C3565 within murine RAW264.7 macrophages. GFP activity was monitored in wild-type CFT073 and its mutants, all of which carried a Pc3566-EGFP fusion plasmid, during infection of RAW264.7 cells. Representative confocal fluorescence micrographs are shown. (TIF) [file ppat.1010005.s009.tif]

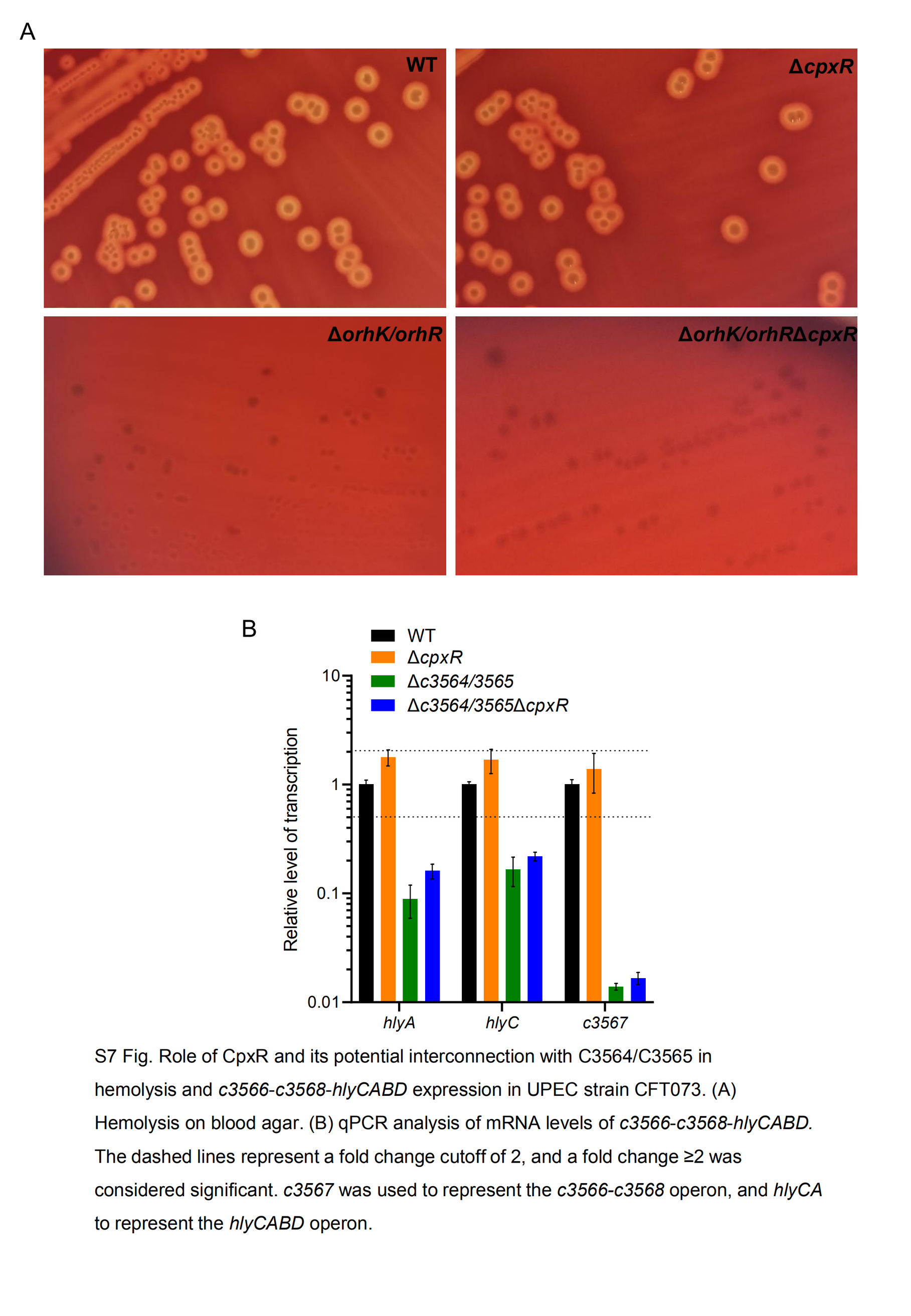

Supplement: S7 Fig — (A) Hemolysis on blood agar. (B) qPCR analysis of mRNA levels of c3566-c3568-hlyCABD. The dashed lines represent a fold change cutoff of 2, and a fold change ≥2 was considered significant. c3567 was used to represent the c3566-c3568 operon, and hlyCA to represent the hlyCABD operon. (TIF) [file ppat.1010005.s010.tif]

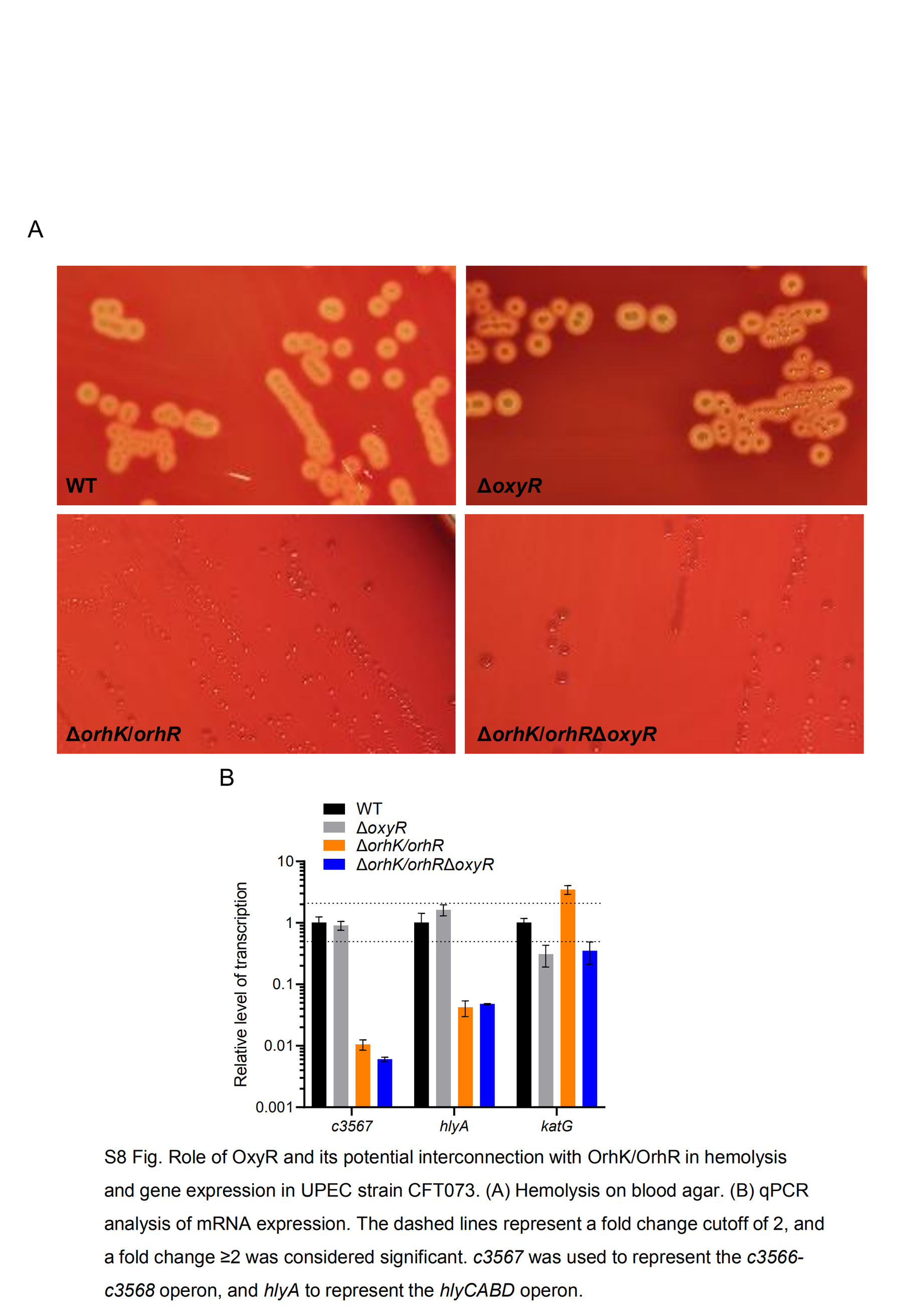

Supplement: S8 Fig — (A) Hemolysis on blood agar. (B) qPCR analysis of mRNA expression. The dashed lines represent a fold change cutoff of 2, and a fold change ≥2 was considered significant. c3567 was used to represent the c3566-c3568 operon, and hlyA to represent the hlyCABD operon. (TIF) [file ppat.1010005.s011.tif]
